# Genome-wide association studies identify the role of caspase-9 in kidney disease

**DOI:** 10.1126/sciadv.abi8051

**Published:** 2021-11-05

**Authors:** Tomohito Doke, Shizheng Huang, Chengxiang Qiu, Xin Sheng, Matthew Seasock, Hongbo Liu, Ziyuan Ma, Matthew Palmer, Katalin Susztak

**Affiliations:** 1 Renal Electrolyte and Hypertension Division, Department of Medicine, University of Pennsylvania, Philadelphia, PA 19104, USA.; 2 Department of Genetics, Perelman School of Medicine, University of Pennsylvania, Philadelphia, PA 19104, USA.; 3 Department of Pathology, Perelman School of Medicine, University of Pennsylvania, Philadelphia, PA 19104, USA.

## Abstract

Genome-wide association studies (GWAS) have identified hundreds of genetic risk regions for kidney dysfunction [estimated glomerular filtration rate (eGFR)]; however, the causal genes, cell types, and pathways are poorly understood. Integration of GWAS and human kidney expression of quantitative trait analysis using Bayesian colocations, transcriptome-wide association studies, and summary-based Mendelian randomization studies prioritized caspase-9 (CASP9) as a kidney disease risk gene. Human kidney single-cell epigenetic and immunostaining studies indicated kidney tubule cells as a disease-causing cell type. Mice with genetic deletion or pharmacological inhibition of CASP9 showed lower apoptosis while having improved mitophagy, resulting in dampened activation of cytosolic nucleotide sensing pathways (cGAS-STING), reduction of inflammation, and protection from acute kidney disease or renal fibrosis. In summary, here, we prioritized CASP9 as an eGFR GWAS target gene and demonstrated the causal role of CASP9 in kidney disease development via improving mitophagy and lowering inflammation and apoptosis.

## Teaser

Integrated omics analysis (GWAS, eQTL, snATAC, and mouse genetic models) identified caspase-9 as a kidney disease risk gene.

## INTRODUCTION

More than 800 million people in the world suffer from kidney disease. Kidney disease is one of the strongest risk factors for cardiovascular death, which can be as high as 15 to 20% annually, once end-stage renal failure is reached (*1*). At present, only a handful of drugs have been approved for the treatment of kidney disease. Currently available therapies slow disease progression but do not stop or reverse kidney function decline. The lack of new therapeutics development is mostly a consequence of poor mechanistic understanding of disease pathogenesis.

Kidney function shows strong heritability; therefore, substantial efforts have been dedicated to identifying the genetic variants associated with this trait (*2*). Genome-wide association studies (GWAS) for kidney function are now available for close to a million participants and identified several hundred loci associated with lower kidney function (*3*–*6*). Almost all GWAS-identified genetic variants are located in the noncoding region of the genome; therefore, disease-causing genes, cell types, and mechanisms remain unknown for the vast majority of these loci.

Several approaches have been taken to understand disease mechanisms based on recent GWAS results. A powerful and popular method has been analyzing the association between risk single-nucleotide polymorphisms (SNPs) and the gene expression of nearby genes, termed expression quantitative trait loci (eQTL) studies (*7*). eQTL studies require genotype and gene expression data for a large number of target tissue samples. Previously, our group has generated a cis-eQTL database for whole-kidney samples, as well as glomeruli and tubuli microdissected from healthy human kidneys of Caucasian individuals (*8*). By integrating results from kidney function GWAS and kidney compartment–specific eQTL studies, we previously prioritized genes for 24 GWAS-identified kidney function–associated risk loci.

Renal tubule epithelial apoptosis has been reported in a variety of different kidney disease models including the cisplatin or ischemia-induced acute kidney injury (AKI) models and the unilateral ureteral obstruction (UUO) kidney fibrosis models (*9*, *10*). Oligomerization of the BCL2-associated X protein (BAX) leads to mitochondrial outer membrane pore formation and cytosolic release of cytochrome c, which is an important upstream regulator of the process. Cytochrome c binds apoptotic peptidase–activating factor 1 (APAF1) and pro–caspase-9 (pro-CASP9), forming an “apoptosome,” resulting in the activation of CASP9 (*11*). CASP9 then cleaves and activates effector caspases such as CASP3 and CASP7. Effector caspases are responsible for the morphological and biochemical changes of apoptosis. While increase in apoptosis has been observed in multiple kidney disease models, the role of apoptosis in kidney disease remains controversial. In early studies, the pan-caspase inhibitor ZVAD-fmk or Bad or Bax knockout mice showed protection from ischemic kidney injury (*12*, *13*). A later study was unable to replicate the protective effect of ZVAD-fmk on kidney function in the ischemic injury model (*14*). Furthermore, in the renal ischemic model, CASP3 knockout mice showed protection from renal fibrosis at later stages but more severe tubule injury at earlier time points (*15*). Likewise, the pan-caspase inhibitor emricasan (IDN-6556) or VX-166, which is shown to attenuate hepatic injury and fibrosis in animal models, failed to meet its targets in clinical studies (*16*, *17*).

There is a complex interaction between apoptosis and other cell death mechanisms such as pyroptosis, ferroptosis, necroptosis, and autophagy (*18*–*21*). Autophagy and apoptosis are the main noninflammatory cell death mechanisms. Depending on the cell type and context, apoptosis can promote or inhibit autophagy. Activated caspases including CASP3 and CASP9 can degrade autophagy proteins such as Beclin1 and ATG5 to inhibit autophagy (*20*, *22*). On the other hand, CASP9 can facilitate autophagosome formation by ATG7 (*23*). Autophagy alteration in renal tubule cells has been observed in cisplatin, UUO, and other kidney disease models (*24*–*27*). Enhanced tubule autophagy is considered to be beneficial by lowering inflammation and by clearing damaged proteins (*28*).

Here, we performed multiomics annotation of the chromosome 1 estimated glomerular filtration rate (eGFR) GWAS locus and prioritized *CASP9* as a likely causal gene. Follow-up mechanistic studies using in vitro and in vivo models demonstrated the role of CASP9 in kidney disease development by regulating not only apoptosis but also autophagy and mitophagy, thereby lowering inflammation and fibrosis.

## RESULTS

### Prioritization of *CASP9* as a kidney disease gene

**Fig. 1.**
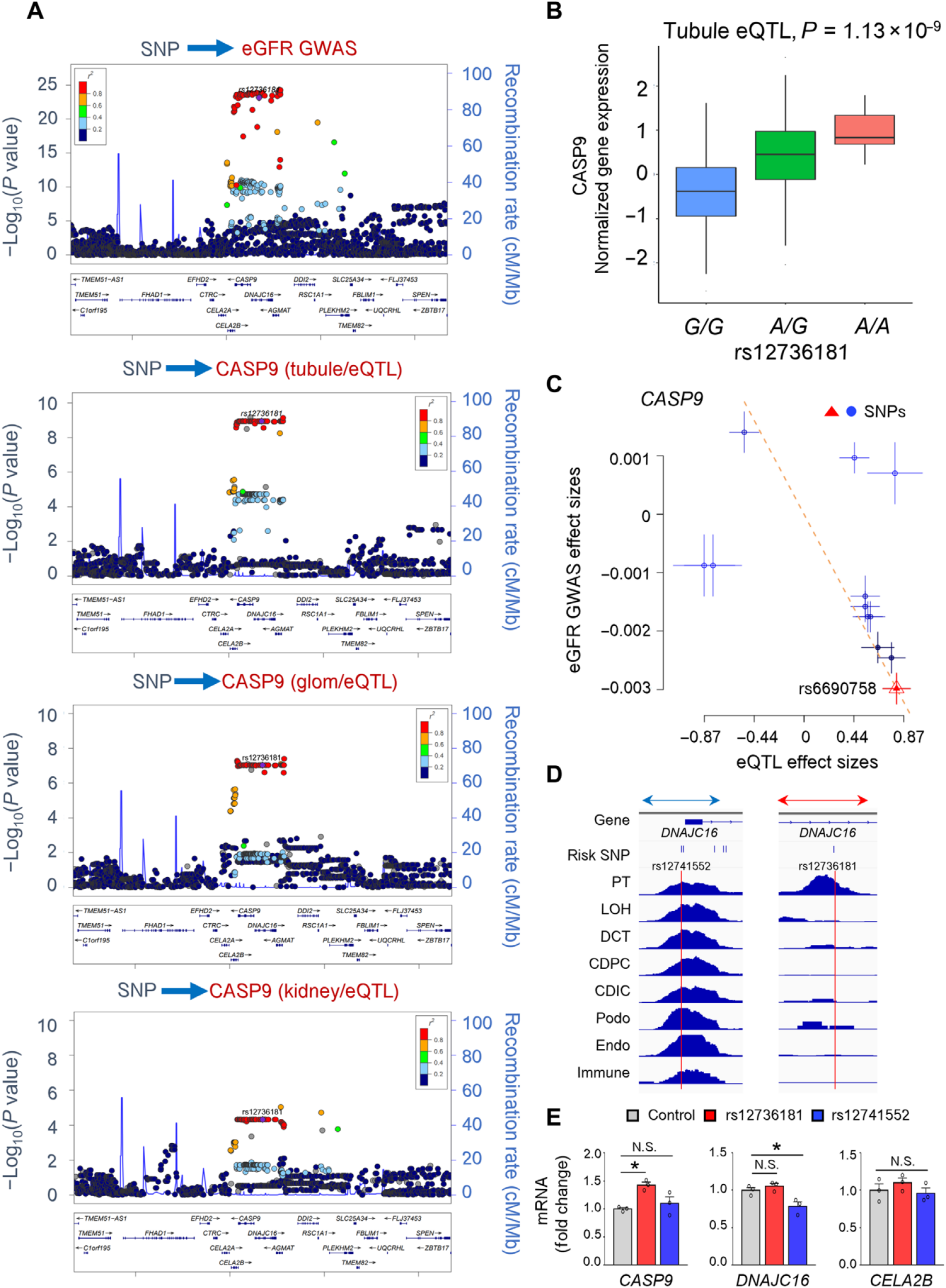
Regional plots of genotype and eGFR (GWAS) and kidney *CASP9* expression (eQTL). (**A**) Locus zoom plots of chromosome 1 region of eGFR GWAS, and *CASP9* eQTL in kidney tubules, glomeruli, and whole kidney. *X* axis shows the chromosomal location of SNPs. *Y* axis shows the strength of association [−log_10_(*P*)]. The data are centered at rs12736181; colors indicate linkage disequilibrium (LD) association. (**B**) Genotype (*x* axis) and relative *CASP9* expression (*y* axis) association in human kidney tubules. (**C**) The effect sizes of SNPs from eGFR GWAS (*y* axis) and eQTL studies (*x* axis). The dashed line represents the SMR estimate, and the red triangle is the rs6690758 that shows strong cis-eQTL effect size. Error bars are the SEs of SNP effects. (**D**) Genome browser view of human kidney snATAC-seq data. The top row shows the gene, followed by the eGFR risk SNPs, and cell type–specific open chromatin information, including proximal tubule (PT), loop of Henle (LOH), distal convoluted tubule (DCT), principal cells of the collecting duct (CDPC), intercalated cells of the collecting tubule (CDIC), podocytes (Podo), endothelial (Endo), and immune cell. The zoom-out version of the region can be viewed in fig. S4. (**E**) The relative mRNA expression levels of *CASP9*, *DNAJC16*, and *CELA2B* in human embryonic kidney (HEK) 293 cells stably expressing Cas9 with or without deletion of the open chromatin region harboring rs12741552 (blue bar) and rs12736181 (red bar) using CRISPR-Cas9 gene editing. The glyceraldehyde-3-phosphate dehydrogenase (GAPDH) was used for internal control. The deleted region was shown on (D) by the blue or red arrows. *n* = 3, technical experimental triplicate. *P < 0.05. N.S., not significant.

In this study, we decided to focus on the chromosome 1 eGFR GWAS locus, which showed a strong and reproducible association with kidney function (Fig. 1A and fig. 1, A to C) (*3*–*6*). Regional plot of eGFR GWAS indicated significant association of genetic variants on chromosome 1 with eGFR (rs12736181, *P* = 7.0 × 10^−24^) (Fig. 1A) (*5*). The same genetic locus also showed significant association with *CASP9* expression in microdissected renal tubules (*P* = 1.1 × 10^−9^), glomeruli (*P* = 9.3 × 10^−8^), and whole-kidney samples (*P* = 9.0 × 10^−6^) (Fig. 1A). Multitissue eQTL analysis performed in GTEx (genotype-tissue expression) showed strong association between genetic variant (rs12736181) and *CASP9* expression levels in multiple tissues including kidney tubules and glomeruli (*m* value, >0.90) (fig. S2 and table S1). On the contrary, we did not observe an association between the genetic variant (rs12736181) and expression of *DNAJC16* and *CLEA2B*, genes located next to *CASP9* (fig. S3, A and B).

To test whether the two traits (eGFR and *CASP9* expression) share causal variants at this locus, we conducted Bayesian colocalization analysis using coloc (*29*). We found strong evidence that variants associated with kidney function and *CASP9* expression in kidney tubules were shared (PP4 = 0.95 and PP3 = 0.05). *CASP9* expression in the kidney were strongly genotype dependent (rs12736181, eGFR GWAS risk allele A, *P* = 1.13 × 10^−9^) (Fig. 1B). Transcriptome-wide association studies (TWAS) and summary-based Mendelian randomization (SMR) (*30*, *31*) indicated that the effect of the genetic variants on eGFR was mediated via *CASP9* expression. In the SMR analysis, eQTL effect sizes negatively correlated with eGFR GWAS effect sizes, suggesting that higher *CASP9* expression was associated with lower eGFR levels (Fig. 1C).

While multiple variants showed association with eGFR and *CASP9* expression, it has been shown that the SNP with the best eQTL effect size or *P* value is not always the causal SNP but, rather, SNPs located in the regulatory region such as enhancer are likely to be causal (*32*, *33*). To address this, we used human kidney single-nuclear assay for transposase-accessible chromatin (snATAC) data to fine-map the region and further prioritize risk variants. Variants that overlapped with open chromatin regions were prioritized. With this prioritization strategy, we narrowed the likely causal SNPs (rs12736181 and rs12741552). Rs12736181 overlapped with open chromatin regions in proximal tubule (PT) cell, while rs12741552 was located on an open chromatin region in all analyzed cell types (Fig. 1D and fig. S4). Both SNPs showed significant association with eGFR and *CASP9* expression, and they were in high linkage disequilibrium (LD) (*r*^2^ = 1).

Next, we performed experiment validation. To examine whether the two prioritized variants regulate the expression of *CASP9*, we deleted open chromatin regions containing these SNPs and examined *CASP9* expression (Fig. 1D). *CASP9* expression was higher upon deleting open chromatin region harboring rs12736181 but unaffected when open chromatin region harboring rs12741552 was deleted. The deletion of the open chromatin region harboring rs12736181 did not affect the expression level of *DNAJC16* and *CELA2B.* The deletion of the open chromatin region harboring rs12741552 did not affect *CELA2B*, while it reduced *DNAJC16* level as it was located on exon1 of *DNAJC16* (Fig. 1, D and E). In summary, integrative genetic analysis, single-nuclear epigenome mapping, and CRISPR-Cas9 gene editing prioritized *CASP9* as a kidney disease risk gene in kidney tubule cells where increased *CASP9* expression was associated with renal disease risk.

### Expression of *Casp9* correlates with fibrosis in a variety of mouse kidney disease models

**Fig. 2.**
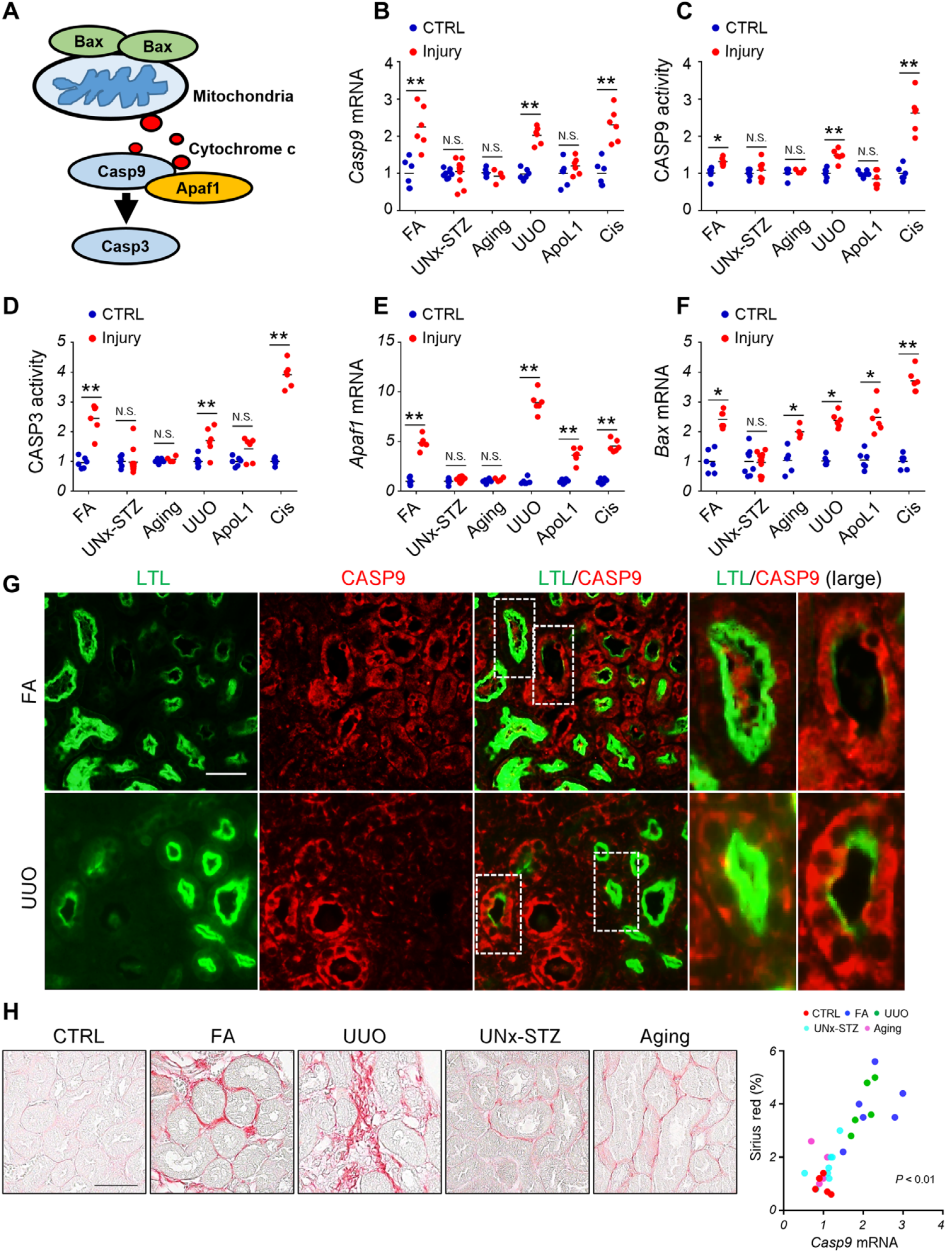
*Casp9* expression and localization in mouse kidney disease models. (**A**) The intrinsic apoptosis pathway. (**B** to **F**) Relative mRNA levels of *Casp9* (B), CASP9 activity (C), CASP3 activity (D), relative mRNA levels of *Apaf1* (E), and *Bax* (F) in kidneys from matched controls (CTRL) and injury models. FA, folic acid; UNx-STZ, uninephrectomy-streptozotocin; UUO, unilateral ureteral obstruction; ApoL1, APOL1 transgenic mice; Cis, cisplatin. (**G**) Representative images of double immunostaining with CASP9 and proximal tubule marker, LTL (*Lotus tetragonolobus* lectin) in FA, and UUO kidneys. Scale bar, 10 μm. The right panels are enlarged images of the highlighted areas. (**H**) Left: Representative images of Sirius red staining of CTRL, FA, UUO, UNx-STZ, and aging mice. Right: The correlation of *Casp9* expression with % area of Sirius red staining in the kidney from CTRL, FA, UUO, UNx-STZ, and aging mice. (B to F) FA CTRL (*n* = 6), FA injury (*n* = 6), UNx-STZ CTRL (*n* = 8), UNx-STZ injury (*n* = 12), aging CTRL (*n* = 6), aging injury (*n* = 4), UUO CTRL (*n* = 6), UUO injury (*n* = 6), ApoL1 CTRL (*n* = 6), ApoL1 transgenic (*n* = 6), Cis CTRL (*n* = 6), and Cis injury (*n* = 6). Data are presented as means ± SEM. *P < 0.05 and **P < 0.01.

While computational integration of multiple genetic studies such as GWAS, eQTL, snATAC, and CRISPR-Cas9 gene editing is an important first step for gene prioritization, experimental validation of target genes is equally critical. CASP9 is the prototypical initiator caspase of intrinsic apoptosis triggered by mitochondrial injury (Fig. 2A) (*11*). We quantified the expression of *Casp9* and other apoptosis-associated genes in a variety of kidney disease models, including the folic acid (FA)–induced kidney fibrosis, the uninephrectomy and streptozotocin (STZ)–induced diabetes (UNx-STZ), renal aging, UUO, podocyte-specific expression of APOL1 risk variant–induced glomerulosclerosis (APOL1) and the cisplatin-induced toxic AKI (Cis) models (*34*–*36*). We found that *Casp9* transcript expression was higher in the FA, UUO, and Cis models (Fig. 2B). CASP9 activity showed consistent changes with transcript levels (Fig. 2C). Furthermore, CASP3 activity and expression of *Apaf1* and *Bax* were also higher in kidneys of FA, UUO, and Cis models, indicating the activation of the entire intrinsic apoptosis cascade in these models (Fig. 2, D to F).

Next, we examined the cellular localization of CASP9 in healthy and disease kidney samples. Immunofluorescence staining showed higher CASP9 expression in FA and UUO fibrosis models compared to healthy control kidneys (Fig. 2G and fig. S5). In addition, double immunofluorescence staining using the proximal tubule marker LTL (*Lotus tetragonolobus* lectin) demonstrated CASP9 expression in renal proximal tubules (Fig. 2G and fig. S5). Tubules with higher CASP9 expression showed weaker LTL signal, indicating that they were likely injured (Fig. 2G). Consistently, we observed CASP9 expression in the proximal tubules in human chronic kidney disease (CKD) kidney samples as assessed by double immunostaining of CASP9 and proximal tubule marker LTL (fig. S6).

To examine the correlation between *Casp9* expression and the degree fibrosis, we next evaluated collagen accumulation in the FA, UUO, UNx-STZ, and aging models of kidney disease. Severe fibrosis was observed in the FA and UUO models, but it was milder in kidneys of the UNx-STZ, and aging models (Fig. 2H). Sirius red–positive area showed a strong correlation with kidney *Casp9* expression (Fig. 2H).

### *Casp9* heterozygous mice were protected from AKI

**Fig. 3.**
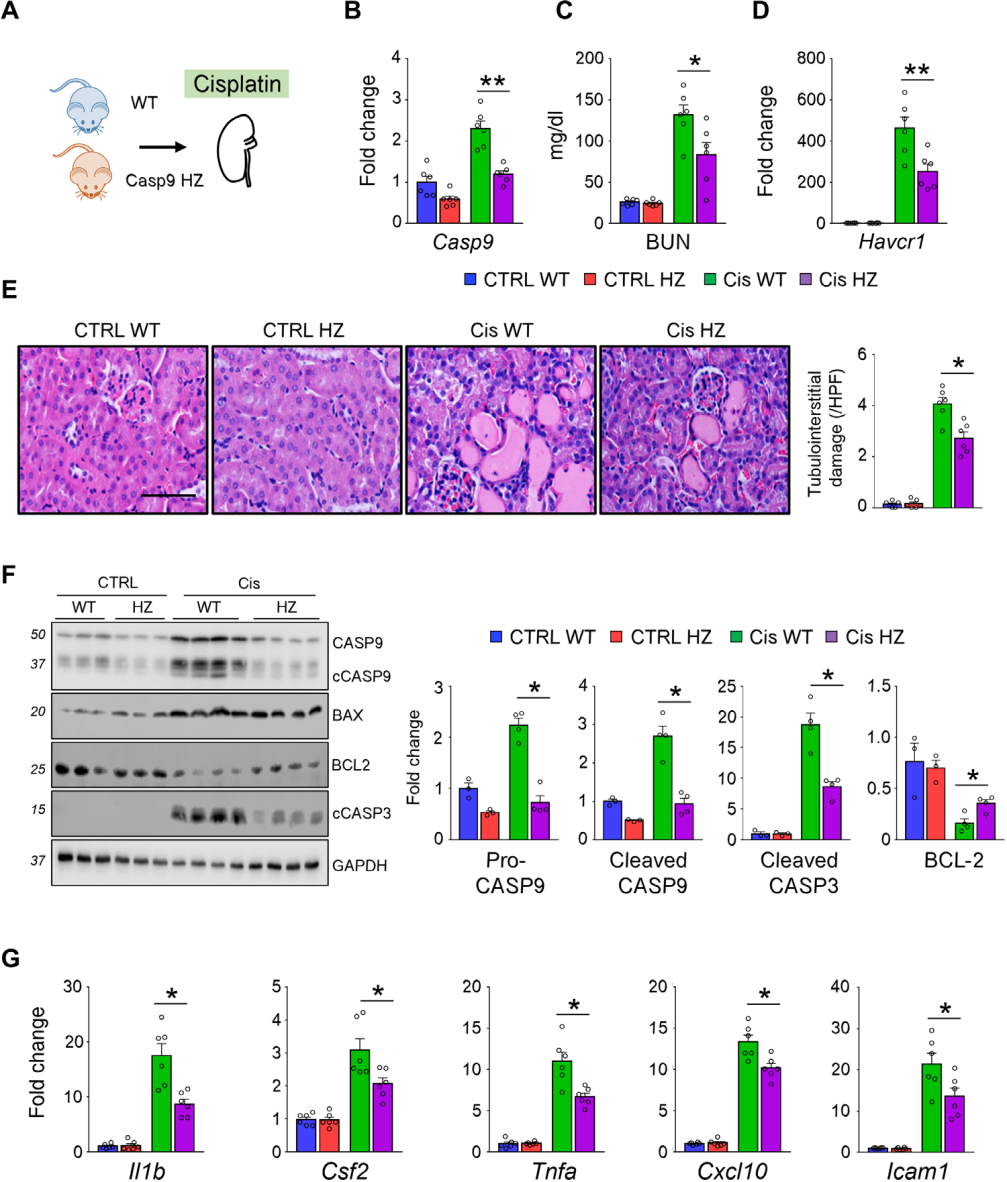
*Casp9* HZ mice are protected from Cis-induced AKI, apoptosis, and inflammation. (**A**) Experiment setup. Wild-type (WT) and *Casp9* HZ (HZ) mice were euthanized 3 days after Cis. CTRL mice were injected with phosphate-buffered saline (PBS). (**B** to **D**) The levels of renal *Casp9* mRNA (B), BUN (C), and renal *Havcr1* mRNA (D) in CTRL and Cis-treated mice. (**E**) Representative images and quantification of tubulointerstitial damage in hematoxylin and eosin sections in CTRL and Cis-injected kidneys of WT and *Casp9* HZ (HZ) mice. Scale bar, 20 μm. (**F**) Western blot image and quantification of pro-CASP9 (CASP9), cleaved CASP9 (cCASP9), BAX, BCL-2, and cleaved CASP3 (cCASP3) in CTRL and Cis-injected kidneys of WT and *Casp9* HZ (HZ) mice. GAPDH was used for loading CTRL. (**G**) Relative expression [quantitative reverse transcription polymerase chain reaction (qRT-PCR)] of *Il1b*, *Csf2*, *Tnfa*, *Cxcl10*, and *Icam1* in CTRL and Cis-injected Cis kidneys of WT and *Casp9* HZ (HZ) mice. CTRL WT (*n* = 6), CTRL HZ (*n* = 6), Cis WT (*n* = 6), and Cis HZ (*n* = 6). Data are presented as means ± SEM. *P < 0.05 and **P < 0.01.

Kidney disease risk allele was associated with a roughly 30% higher *CASP9* expression in human kidney tubule samples. We analyzed *Casp9* heterozygous (*Casp9* HZ) mice, with about 50% change in *Casp9* expression (Fig. 3A) (*37*). At baseline, we did not observe significant differences in survival, body weight, serum electrolyte, and kidney function parameters such as blood urea nitrogen (BUN) between wild-type (WT) and *Casp9* HZ mice at 6 and 24 months of age (table S2).

We next analyzed changes in injury setting, such as induced by cisplatin injection. The kidney function marker BUN was lower in *Casp9* HZ when compared to WT mice after cisplatin injection (Fig. 3, A to C). The hepatitis A virus cellular receptor 1 (*Havcr1* or *Kim1*) expression, a typical renal tubular injury marker, was markedly lower in kidneys of cisplatin-treated *Casp9* HZ mice (Fig. 3D). Kidneys of cisplatin-treated WT mice showed marked tubule epithelial damage, such as loss of brush border, epithelial cell death, tubular dilation, tubular cast, and infiltration of inflammatory cells (Fig. 3E). Renal injury score was lower in cisplatin-treated *Casp9* HZ compared to cisplatin-treated WT mice (Fig. 3E). The expression levels of apoptosis markers such as cleaved CASP9 and cleaved CASP3 were lower, and BCL2 was higher in cisplatin-treated kidneys of *Casp9* HZ compared to WT mice (Fig. 3F and fig. S7A). Similarly, cultured *Casp9* HZ renal tubule cells showed lower expression of apoptotic caspases and improved viability following cisplatin treatment (fig. S8, A to C). Although apoptosis per se is a noninflammatory cell death mechanism, expression of cytokines (*Il1b*, *Csf2*, *Tnfa*, and *Cxcl10*) and *Icam1* was also markedly lower in kidneys of cisplatin-injected *Casp9* HZ mice compared to cisplatin-injected WT mice (Fig. 3G).

### Pharmacological inhibition of CASP9 ameliorated cisplatin-induced AKI

**Fig. 4.**
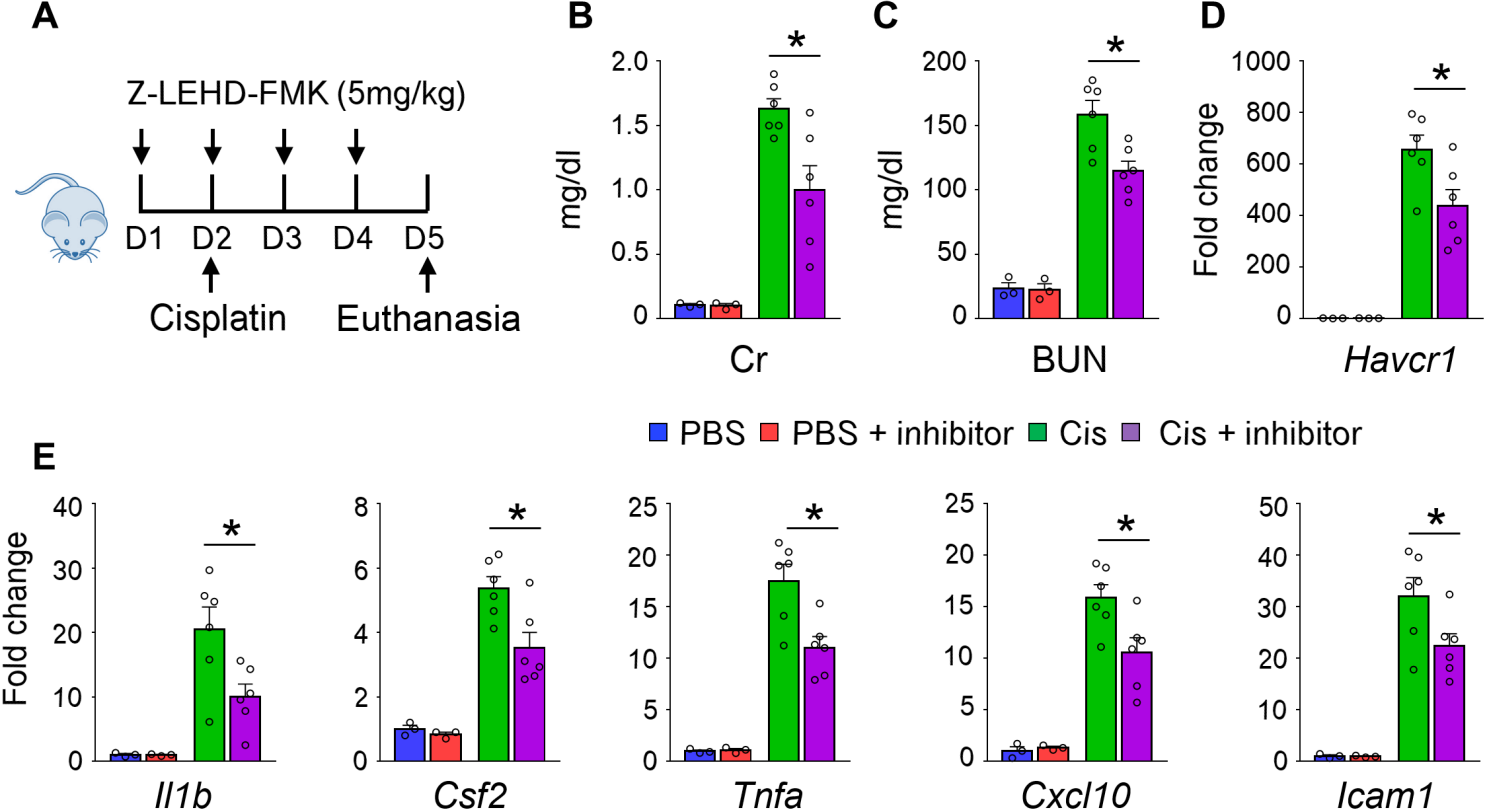
Pharmacological inhibition of CASP9 protected from Cis-induced kidney injury and inflammation. (**A**) Experimental design. (**B** to **D**) The levels of serum creatinine (Cr) (B), BUN (C), and renal *Havcr1* mRNA (D) in PBS-injected (PBS) or Cis-injected mice with CASP9 inhibitor (inhibitor) or vehicle CTRL. (**E**) Relative renal expression (qRT-PCR) of *Il1b*, *Csf2*, *Tnfa*, *Cxcl10*, and *Icam1*. PBS with vehicle CTRL (*n* = 3), PBS with CASP9-specific inhibitor (*n* = 3), Cis with vehicle CTRL (*n* = 6), and Cis with CASP9-specific inhibitor (*n* = 6). Data are presented as means ± SEM. *P < 0.05.

Several small molecular inhibitors that target CASP9 have been developed with good specificity (*38*, *39*). We investigated the effect of Z-LEHD-FMK (a CASP9 inhibitor) in the cisplatin model of kidney injury. Mice were injected with CASP9 inhibitor or vehicle daily starting 1 day before cisplatin administration. Serum creatinine and BUN levels were lower, and renal expression of *Havcr1* was dampened in Z-LEHD-FMK–treated mice. Furthermore, we also observed that inflammatory cytokines (*Il1b*, *Csf2*, *Tnfa*, and *Cxcl10*) and *Icam1* expression were lower in inhibitor-treated mice after cisplatin injury (Fig. 4, A to E). Collectively, we found improved kidney function and lessened kidney damage in the cisplatin-injected mice treated with Z-LEHD-FMK.

### Enhanced autophagy in cisplatin-treated *Casp9* HZ mice

**Fig. 5.**
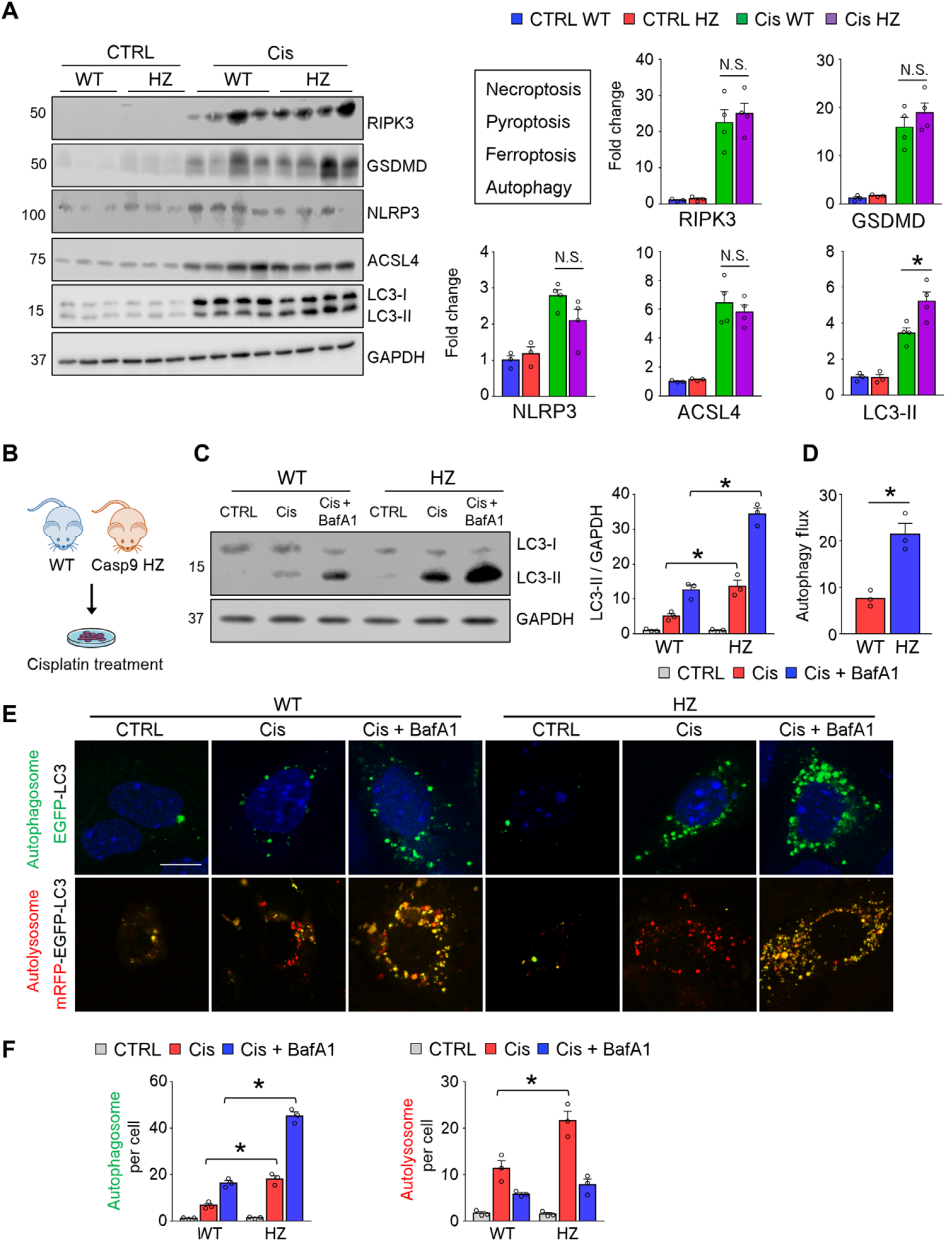
Improved autophagy flux in Cis-treated *Casp9* HZ renal tubule cells. (**A**) Western blot image and quantification of RIPK3, GSDMD, NLRP3, ACSL4, and LC3-II in CTRL and Cis-treated kidneys from WT and *Casp9* HZ (HZ) mice. GAPDH was used for loading CTRL. (**B**) Isolation of renal tubule cells from WT and *Casp9* HZ (HZ) mice. (**C**) Representative Western blot and quantification of LC3-II in cultured renal tubule cells from WT and *Casp9* HZ (HZ) mice in indicated conditions (Cis + BafA1, Cis with bafilomycin A1). Cells were exposed to Cis for 8 hours. GAPDH was used for loading CTRL. (**D**) Autophagy flux in WT and *Casp9* HZ (HZ) renal tubule cells. Flux was calculated as a difference in LC3-II levels (corrected by GAPDH) between Cis and Cis + BafA1 group. Three independent experiments were performed. (**E**) Representative confocal image of the renal tubule cell transfected with EGFP-LC3 plasmid or mRFP-EGFP-LC3 plasmid in indicated conditions. Scale bar, 10 μm. (**F**) Quantification of autophagosome (left) and autolysosome (right) in the renal tubule cell in indicated conditions. Experiments were performed three times, and in each experiment, *n* = 10 cells were counted and mean value was plotted in the graph. Data are presented as means ± SEM. *P < 0.05.

The role of apoptosis in kidney disease development is controversial. While lower apoptosis is associated with cellular preservation, absence of apoptosis can divert to more inflammatory cell death pathways. We were surprised to find that both genetic and pharmacological inhibition of CASP9 was associated with dampened inflammation; therefore, we decided to investigate changes in inflammatory cell death pathways such as necroptosis, pyroptosis, and ferroptosis (*40*). The expression levels of critical regulators of necroptosis (RIPK3), pyroptosis [NLRP3 and gasdermin D (GSDMD)], and ferroptosis [acyl–coenzyme A synthetase long-chain family member 4 (ACSL4)] were higher in kidneys of cisplatin-injected mice. On the other hand, we did not observe differences in inflammatory cell death pathways between cisplatin-treated WT and *Casp9* HZ mice (Fig. 5A).

At the same time, the expression levels of LC3-II, a key molecule in autophagy, were higher in cisplatin-treated kidneys of WT mice, and it was further increased in cisplatin-treated kidneys of *Casp9* HZ mice (Fig. 5A). Therefore, we next analyzed autophagy markers in renal tubule cells isolated from WT and *Casp9* HZ mice at baseline and following cisplatin treatment. Autophagy flux was examined by treating cells with bafilomycin A1 (BafA1), which interferes with lysosomal function halting autophagosome-lysosomal fusion, enabling the quantification of the rate of autophagy. The LC3-II expression was higher in *Casp9* HZ renal tubule cells exposed to cisplatin, and its level further increased following BafA1 treatment (Fig. 5, B and C) indicating an improved autophagy flux in cisplatin-treated *Casp9* HZ renal tubule cells (Fig. 5D). To better understand changes in autophagy, we transfected renal tubule cells with enhanced green fluorescent protein (EGFP)–LC3 plasmid. Autophagosome number, represented by EGFP-positive dots, was markedly higher in *Casp9* HZ renal tubule cells exposed to cisplatin, which again further increased following BafA1 treatment (Fig. 5, E and F). The mRFP-EGFP-LC3 plasmid is another tool to evaluate autophagy flux by using different pH stability of GFP and red fluorescent protein (RFP). Under acidic conditions such as autolysosome, the GFP signal is quenched and only the RFP signal is visible. Autolysosome numbers indicated by RFP puncta were observably higher in *Casp9* HZ renal tubule cells exposed to cisplatin (Fig. 5, E and F). BafA1 treatment restored the GFP signal.

### Suppression of the cytosolic nucleotide sensing pathways (cGAS and STING) by mitophagy

**Fig. 6.**
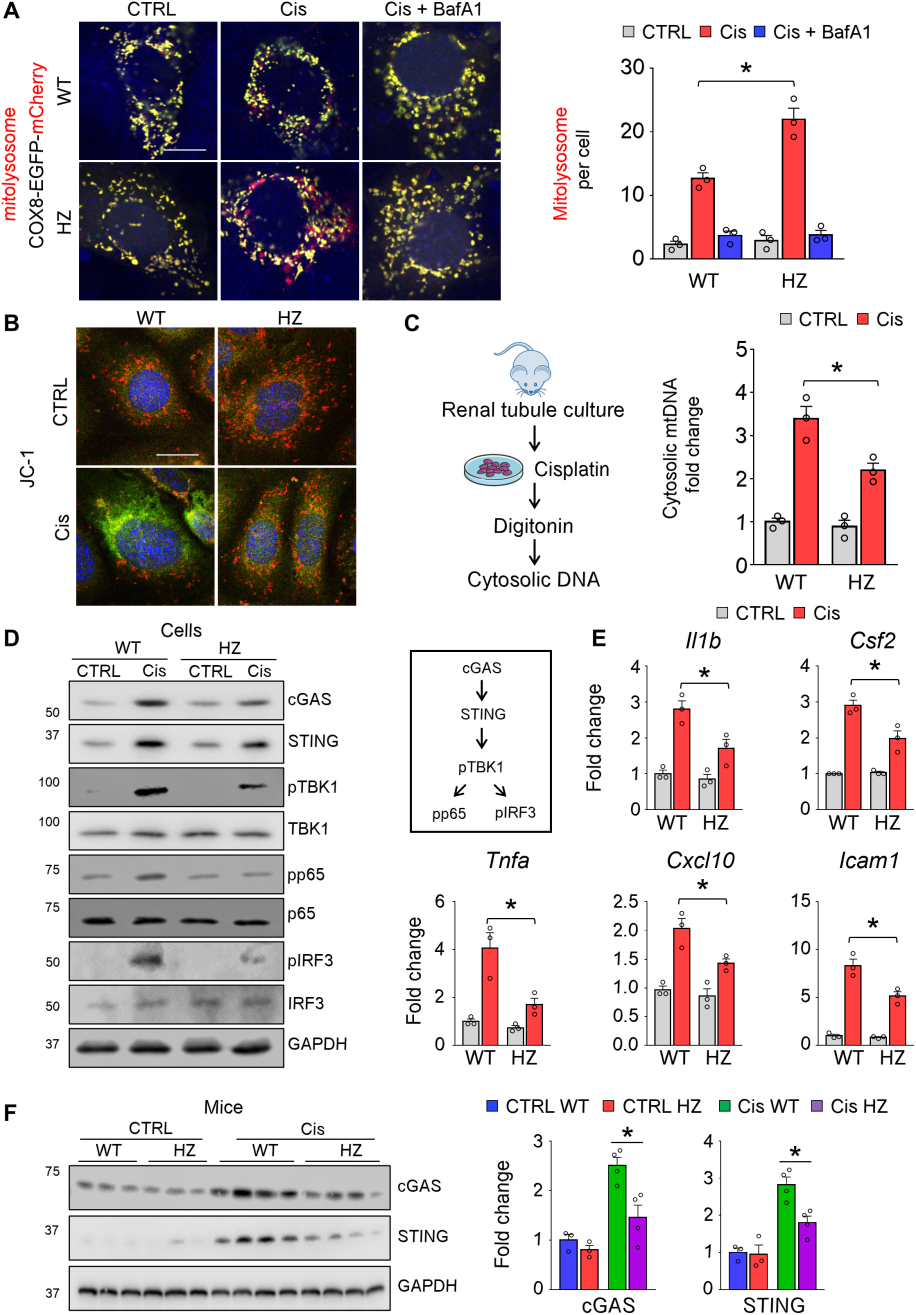
Improved mitophagy and lower cGAS-STING activation in Cis-treated *Casp9* HZ renal tubule cells. (**A**) Representative confocal images of renal tubule cells transfected with COX8-EGFP-mCherry plasmid. Quantification of mCherry dots (mitolysosome) in indicated conditions. Scale bar, 10 μm. Three independent experiments were performed. In each experiment, *n* = 10 cells were counted and mean value was plotted. (**B**) Representative confocal image of JC-1–stained Cis or PBS-treated (CTRL) WT and *Casp9* HZ (HZ) renal tubule cells. Scale bar, 10 μm. Green fluorescence intensity indicates mitochondrial depolarization. (**C**) Extraction and quantification of cytosolic mitochondrial DNA (mtDNA) of Cis or PBS-treated (CTRL) WT and *Casp9* HZ (HZ) renal tubule cells. (**D**) Representative Western blot image of cGAS, STING, pTBK1, TBK1, pp65, p65, pIRF3, and IRF3 in Cis- or PBS-treated (CTRL) WT and *Casp9* HZ (HZ) renal tubule cells. (**E**) Relative expression (qRT-PCR) of *Il1b*, *Csf2*, *Tnfa*, *Cxcl10*, and *Icam1* in Cis- or PBS-treated (CTRL) WT and *Casp9* HZ (HZ) renal tubule cells. (**F**) Western blot image and quantification of cGAS and STING in CTRL and Cis kidneys of WT and *Casp9* HZ (HZ) mice. GAPDH was used for loading CTRL. Data are presented as means ± SEM. *P < 0.05.

As we observed changes in autophagy, we next analyzed mitophagy using COX8-EGFP-mCherry plasmid. While cisplatin induced a severe mitophagy defect, mitolysosomes represented by the mCherry signal were markedly higher in *Casp9* HZ renal tubule cells exposed to cisplatin (Fig. 6A). Cisplatin induced marked mitochondrial depolarization in kidney tubule cells, which was ameliorated in *Casp9* HZ renal tubule cells (Fig. 6B). The cisplatin-induced mitochondrial damage has been shown to release mitochondrial DNA, which is sensed by the cytosolic nucleotide sensing pathways such as cGAS (cyclic GMP–AMP synthase) leading to STING (stimulator of interferon genes) activation (*41*). We found that the cytosolic mitochondrial DNA release was lower in *Casp9* HZ renal tubule cells when compared to WT following cisplatin treatment (Fig. 6C). Subsequently, protein levels of cGAS and STING were lower in cisplatin-treated *Casp9* HZ renal tubule cells. Downstream signaling molecules, such as phosphorylated TANK-binding kinase 1 (pTBK1), phosphorylated p65 (pp65), and phosphorylated interferon regulatory factor 3 (pIRF3) were lower in cisplatin-treated *Casp9* HZ renal tubule cells (Fig. 6, D and E, and fig. S9A). Furthermore, expression of downstream proinflammatory cytokines such as *Il1b*, *Csf2*, *Tnfa*, *Cxcl10*, and *Icam1* was also lower in cisplatin-treated *Casp9* HZ renal tubule cells (Fig. 6F).

Last, we validated changes observed in cytosolic nucleotide sensing pathways in kidneys of cisplatin-treated WT and *Casp9* HZ mice. Protein levels of cGAS and STING were also lower in cisplatin-treated *Casp9* HZ mice when compared to cisplatin-treated WT mice (Fig. 6G).

### *Casp9* HZ mice are protected from kidney fibrosis

**Fig. 7.**
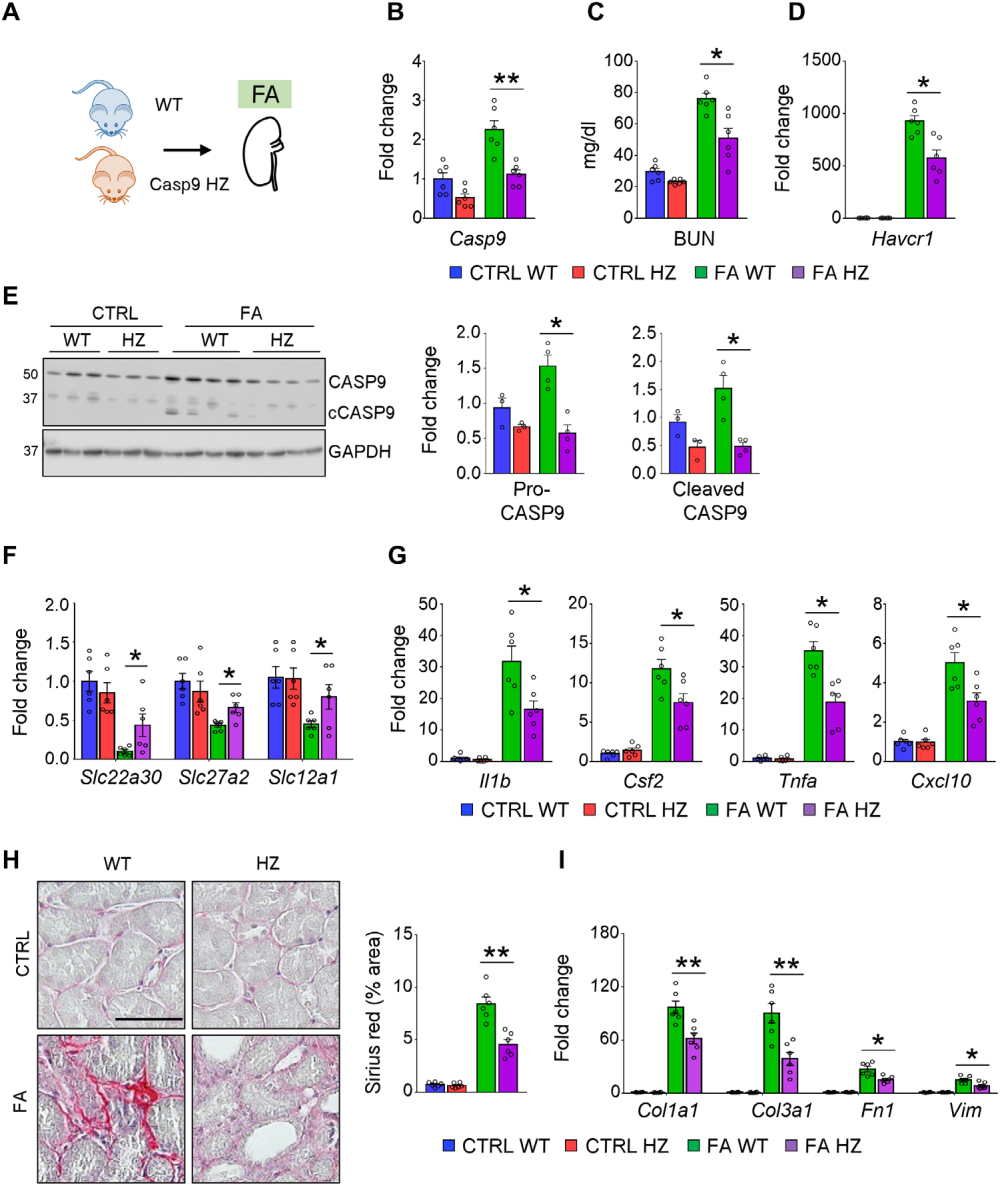
*Casp9* HZ mice are protected from FA-induced kidney fibrosis. (**A**) WT and *Casp9* HZ (HZ) mice were euthanized 7 days after FA injection (intraperitoneal injection of 250 mg/kg FA). (**B** to **D**) The levels of renal *Casp9* mRNA (B), BUN (C), and renal *Havcr1* mRNA (D) in CTRL and FA-induced mice. (**E**) Western blot image and quantification of pro-CASP9 (CASP9), cleaved CASP9 (cCASP9) in CTRL, and FA-induced kidneys from WT and *Casp9* HZ (HZ) mice. GAPDH was used for loading CTRL. (**F** and **G**) Relative expression (qRT-PCR) of *Slc22a30*, *Slc27a2*, *Slc12a2* (F), *Il1b*, *Csf2*, *Tnfa*, *Cxcl10*, and *Icam1* (G) in CTRL and Cis-induced kidneys from WT and *Casp9* HZ (HZ) mice. (**H**) Representative images and quantification of tubulointerstitial fibrosis by Sirius red staining in CTRL and FA-induced kidneys from WT and *Casp9* HZ (HZ) mice. Scale bar, 20 μm. (**I**) Relative expression (qRT-PCR) of *Col1a1*, *Col1a3*, *Fn1*, and *Vim* in CTRL and FA-induced kidneys from WT and *Casp9* HZ (HZ) mice. CTRL WT (*n* = 6), CTRL HZ (*n* = 6), FA WT (*n* = 6), and FA HZ (*n* = 6). Data are presented as means ± SEM. *P < 0.05 and **P < 0.01.

While our studies using the cisplatin model demonstrated the role of CASP9 in AKI, we next examined whether CASP9 plays a role in kidney fibrosis, a feature of CKD. We evaluated the FA injection crystal precipitation model as we found earlier an increase in kidney *Casp9* levels in this model (Figs. 2B and 7, A and B). We found that markers of kidney function (BUN) and expression of tubule injury markers (*Havcr1*) were improved in FA-treated *CASP9* HZ mice (Fig. 7, C and D). We observed an increase in expression of proapoptotic proteins such as CASP9 and cleaved CASP3 in kidneys of FA-treated mice, which were attenuated in *Casp9* HZ mice (Fig. 7E and fig. S7B). Consistent with the lower apoptosis rate, expression of proximal and distal tubule marker genes (*Slc22a30*, *Slc27a2*, and *Slc12a1*) were preserved in the FA-treated *Casp9* HZ mice (Fig. 7F). At the same time, expression of inflammatory cytokines (*Il1b*, *Csf2*, *Tnfa*, and *Cxcl10*), renal fibrosis, and profibrotic genes (*Col1a1*, *Col3a1*, *Fn1*, and *Vim*) was lower in the FA-treated *Casp9* HZ mice (Fig. 7, G to I).

**Fig. 8.**
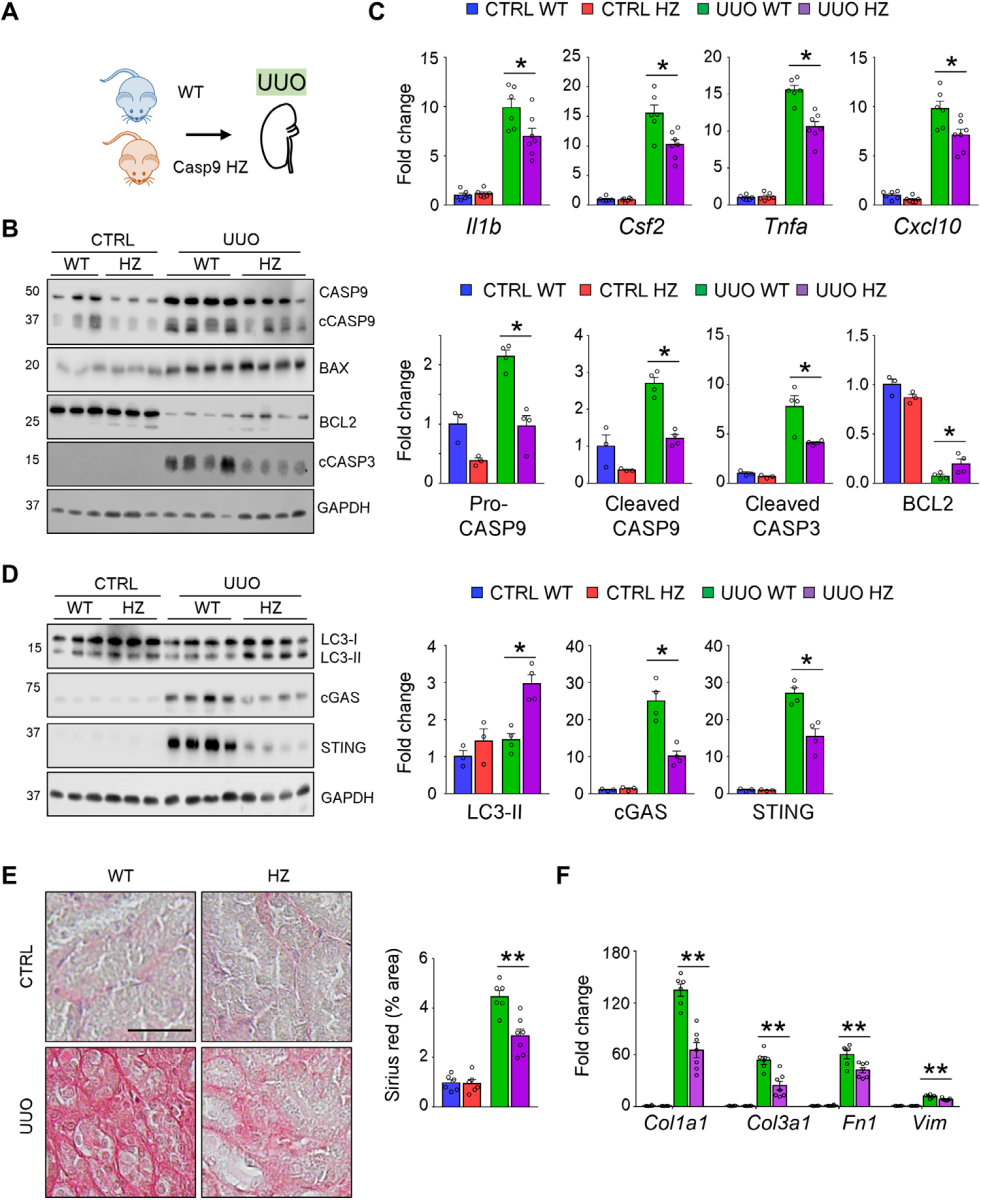
Lower inflammation and kidney fibrosis in *Casp9* HZ mice subjected to UUO injury. (**A**) Experimental design. (**B**) Western blot image and quantification of pro-CASP9 (CASP9), cleaved CASP9 (cCASP9), BAX, BCL2, and cleaved CASP3 (cCASP3) in CTRL and UUO kidney from WT and *Casp9* HZ (HZ) mice. GAPDH was used for loading CTRL. (**C**) Relative expression (qRT-PCR) of *Il1b*, *Csf2*, *Tnfa*, and *Cxcl10* in CTRL and UUO kidney from WT and *Casp9* HZ (HZ) mice. (**D**) Western blot image and quantification of LC3-II, cGAS, and STING in CTRL and UUO kidney from WT and *Casp9* HZ (HZ) mice. GAPDH was used for loading CTRL. (**E**) Representative images and quantification of tubulointerstitial fibrosis by Sirius red staining in CTRL and UUO kidney from WT and *Casp9* HZ (HZ) mice. Scale bar, 20 μm. (**F**) Relative transcript expression (qRT-PCR) of *Col1a1*, *Col1a3*, *Fn1*, and *Vim* in CTRL and UUO kidney from WT and *Casp9* HZ (HZ) mice. CTRL WT (*n* = 6), CTRL HZ (*n* = 6), UUO WT (*n* = 6), and UUO HZ (*n* = 7). Data are presented as means ± SEM. *P < 0.05 and **P < 0.01.

Similarly, we observed less tubule injury and marked differences in apoptosis in *Casp9* HZ mice following UUO (Fig. 8, A and B and fig. S10A). The expression of inflammatory cytokine levels (*Il1b*, *Csf2*, *Tnfa*, and *Cxcl10*) was lower in UUO kidneys from *Casp9* HZ mice (Fig. 8C). We did not observe differences in necroptosis, pyroptosis, and ferroptosis regulators (examined by RIPK3, GSDMD, NLRP3, and ACSL4) in UUO kidneys when *Casp9* HZ and WT mice were compared (fig. S11A). The higher LC3-II levels in UUO kidney of *Casp9* HZ mice were consistent with improved autophagy (Fig. 8D). Kidney expression of cGAS and STING was lower in UUO *Casp9* HZ mice (Fig. 8D). Last, the lesser inflammation was associated with lower renal fibrosis and expression of profibrotic genes (*Col1a1*, *Col3a1*, *Fn1*, and *Vim*) in UUO kidney of *Casp9* HZ mice (Fig. 8, E and F).

## DISCUSSION

Here, we demonstrate that *CASP9* is a kidney disease risk gene prioritized by a variety of multiomics approaches. Follow-up in vitro and in vivo studies indicate that CASP9 not only lowered renal tubule cell apoptosis but also improved mitophagy, resulting in the reduction of cytosolic mitochondrial DNA, cytosolic nucleotide sensing pathways (cGAS and STING), downstream inflammation, and fibrosis development.

While GWAS identifies risk regions in the genome, orthogonal datasets are needed for the functional interpretation of GWAS risk variants, such as kidney eQTL and single-nuclear epigenetic information. Bayesian colocalization analysis revealed that the GWAS risk variants had a shared effect on both eGFR and kidney tubule *CASP9* expression levels, where higher *CASP9* expression levels were associated with lower eGFR levels. SMR and TWAS analysis indicated that *CASP9* expression mediated the effect of the genotype on kidney function. Integration of Bayesian colocalization signal with single-nuclei open chromatin information prioritized likely causal variant and indicated tissue-specific (kidney), and cell type–specific (tubules) regulation of *CASP9* expression. CRISPR-Cas9 gene editing confirmed the functional role of the open chromatin region containing likely causal variants.

Here, we show that kidney *Casp9* mRNA expression levels correlated with fibrosis severity. Higher *Casp9* expression and activity and more severe fibrosis were observed in the FA and UUO models compared to UNx-STZ and aging mice. The FA and UUO models are widely recognized as kidney fibrosis models (*42*–*45*), while the diabetic and aging models only show mild histological changes and lack of GFR decline (*46*–*48*). CASP9 expression in proximal tubules of FA and UUO mice and patients with CKD supports the key role of proximal tubule in kidney disease development.

Via the use of mouse genetic models, we demonstrated the causal role of *CASP9* in AKI, fibrosis, and inflammation, rendering *CASP9* a kidney disease risk gene. Genetic deletion or pharmacological inhibitor of CASP9 lowered apoptosis and protected from acute injury induced by cisplatin and kidney fibrosis induced by FA and UUO. The protective role of CASP9 in kidney disease development can be explained by two mechanisms. First, apoptosis seems to contribute to tubule epithelial cell loss, such as proximal tubule and the loop of Henle cells. Preserved epithelial cell number likely results in functional preservation; however, as epithelial cells can regenerate, this might not fully explain the protection from disease development.

Second, our observations indicate that mechanisms outside of apoptosis play an important role in the CASP9-afforded protection. The *Casp9* expression and CASP9 activity did not fully correlate with classic apoptosis genes such as *Bax* and *Apaf1* levels. We observed improved mitophagy in *Casp9* HZ renal tubule cells treated with cisplatin and UUO model of kidney fibrosis. Defect in mitophagy has been associated with cytosolic leakage of mitochondrial DNA and activation of the cytosolic nucleotide sensing pathways following subsequent inflammation (*49*). We observed marked differences in cGAS and STING activation and lower inflammation in *Casp9* HZ renal tubule cells and mice. These observations are consistent with previous reports showing the pathogenic proinflammatory role of the cGAS-STING pathway in kidney fibrosis (*50*).

Future studies shall examine the molecular mechanism of the interaction between CASP9 and autophagy/mitophagy. Experimental demonstration of the association of improved autophagy/mitophagy in tubule epithelial with renal inflammation will also be important.

In summary, we prioritized *CASP9* as an eGFR GWAS target gene using computational and experimental tools, as well as cellular and animal models. We demonstrate the causal role of CASP9 in kidney disease development via improving mitophagy and lowering inflammation and apoptosis. Our results could open new avenues for kidney disease therapeutics via the use of small molecular CASP9 inhibitors.

## METHODS

### Visualization of SNPs in GWAS and eQTL

LocusZoom (*51*) was used to view genomic regions of interest in chromosome 1 for kidney function GWAS (*3*–*6*) and eQTL of *CASP9*, *CELA2B*, and *DNAJC16* in tubules, glomeruli, and whole kidneys (*8*). eQTL box plots were used to show the associations between reference and alternative alleles of given genotypes and *CASP9* expression in tubule samples.

### Colocalization analysis

The eQTL dataset of tubules, glomeruli, and whole kidney has been generated as previously published (*8*). To estimate the posterior probabilities of sharing the same genetic variants between kidney function and gene expression in tubuli and glomeruli, we performed colocalization analysis using coloc (*29*) and GWAS and eQTL summary data (*5*). A 100-kilobase region around the index SNP was used to calculate posterior probability. In the coloc results, H3 represents the posterior probability that both traits (kidney function and gene expression) are associated, but with different causal variants; H4 represents the posterior probability that both traits are associated and share a single causal variant. PP_H4 > 0.8 was used for the threshold of colocalization.

### TWAS FUSION

TWAS FUSION pipeline was performed using eGFR GWAS (*5*) and tubule eQTL data (*8*) as previously described (*30*, *52*). We generated gene expression weights using human kidney tubule RNA sequencing (*n* = 121) data following the FUSION pipeline (*30*). Genotypes were imputed to the 1000 Genomes Phase 1 v3, and restricted to well-imputed (information score, >0.9) sites. Reads per kilobase of transcript per million mapped reads and log-adjusted gene expression levels were estimated in a generalized linear model controlling for three gene expression principal components and rank-normalized. We filtered genes that did not exhibit cis-genetic regulation at current samples sizes by keeping only genes with nominally significant estimates of cis-SNP heritability. We refrained from reporting genes in the human leukocyte antigen region due to the complicated LD patterns. To generate predictive models, FUSION defines gene expression for samples as a linear function of SNPs in a 1-megabase region flanking the gene as where are the SNP weights, are covariates (e.g., sex, age, genotype principal components, genotyping platform, PEER factors) and their effects, and is random environmental noise (*53*). FUSION estimated weights for expression of a gene in a tissue using multiple penalized linear models (here, we used LASSO).

### Summary-based Mendelian randomization

SMR was used to test potential causal effects of a gene on complex trait, given an SNP as an instrumental variable (*31*), using summary-level data from eGFR GWAS (*5*) and eQTL studies (*8*). SMR reports the association for visualization of SMR analysis, and we depicted SMR effect plot with SMREffectPlot function in plot_SMR.r using multiple variants in the cis-eQTL region of genes.

### Multiple-tissue eQTL mapping

The eQTL summary results of 44 other human tissues were downloaded from GTEx (*54*). METASOFT (*55*), a meta-analysis method, was performed on all variant-gene pairs that were significant (false discovery rate < 5%) in at least 1 of the 46 tissues (two kidney compartments; tubules and glomeruli and 44 GTEx tissues). A random-effects model in METASOFT (called RE2) was used, and the posterior probability (*m* value) was calculated for each SNP-gene pair and tissue tested. The significance cutoff of *m* > 0.9 was used to discover high-confidence eQTLs.

### Human kidney snATAC sequencing

Human kidneys were homogenized and single-nuclear suspension for snATAC sequencing (snATACseq) was prepared according to the manufacturer’s protocol (10X Genomics) as previously described (*56*, *57*). Quality control for constructed library was perform by an Agilent Bioanalyzer High Sensitivity DNA kit. The libraries were sequenced on an Illumina HiSeq. The data processing and analysis were performed as previously described (*57*).

### CRISPR-Cas9 genomic deletions

Human embryonic kidney (HEK) 293 cells stable expressing Cas9 was a gift of L. Wang and L. Song (University of Pennsylvania). Guide RNAs were designed by CRISPOR and cloned into pLKO.sgRNA plasmid. After confirming insert sequences, plasmids were transfected into Cas9-expressing HEK293 using Lipofectamine 3000 (Thermo Fisher Scientific, #L3000015) at 70 to 80% confluent according to the manufacturer’s instruction. Cultured cells were collected 48 hours after transfection, and RNA was extracted using TRIzol as previously described (*52*). Gene expression was quantified by quantitative reverse transcription polymerase chain reaction (qRT-PCR). Target genomic region deletion was confirmed by Sanger sequencing. The full guide RNA sequences were included in table S3.

### SNP prioritization

The likely causal SNP was prioritized by the following method: (i) reached genome-wide significance in eGFRcrea GWAS (*5*), (ii) reached genome-wide significance in human kidney eQTL for *CASP9* (*8*), (iii) located in an open chromatin area in human kidney snATACseq data (*57*), and (iv) altered the expression of the target gene in CRISPR-Cas9 locus deletion experiments.

### Sample procurement

Human kidney samples were obtained from surgical nephrectomies. Nephrectomies were deidentified, and the corresponding clinical information was collected through an honest broker; therefore, no consent was obtained from the individuals.

### Mouse studies

Eight- to 10-week-old male WT mice and littermate male *Casp9* HZ mice were used for the cisplatin, FA, and UUO models. Casp9 HZ mice were obtained from R. A. Flavell in the Yale University (*37*). Mice were injected intraperitoneally with FA (#10752485 ACROS Organics, 250 mg/kg, dissolved in 300 mM sodium bicarbonate) or sodium bicarbonate (#S6014, Sigma-Aldrich) and euthanized on day 7. Mice were euthanized on day 7 after left ureteral ligation or sham surgery. Mice were euthanized on day 3 after administration of a single dose of cisplatin (20 mg/kg body weight) (Cayman, #Cay13119) or phosphate-buffered saline (PBS). Mice were euthanized 18 weeks after uninephrectomy and following low-dose STZ (50 mg/kg, i.p. for 5 days) (streptozotocin, Santa Cruz Biotechnology, #U-9889) or PBS injection. Aging mice were euthanized at 2 years of age. Transgenic mice (Nphs1-rtTA/TRE-APOL1-G2) were placed on a doxycycline diet to induce transgene expression and euthanized as described previously (*35*). All animal experiments were approved by the University of Pennsylvania ethics committee (Institutional Animal Care and Use Committee).

### Quantitative reverse transcription polymerase chain reaction

RNA was isolated from kidney tissue or cells using TRIzol (Invitrogen, #15596018). RNA was reverse-transcribed using the High-Capacity cDNA Reverse Transcription Kit (Applied Biosystems, #4368813), and qRT-PCR was run in the ViiA 7 System (Life Technologies) instrument using SYBR Green Master Mix (Applied Biosystems, #4367659) and gene-specific primers. For quantitative analysis, samples were normalized to glyceraldehyde-3-phosphate dehydrogenase (GAPDH) with the ΔΔ*C*_t_ method. Primer sequences are listed in table S4.

### Western blot

Tissue lysates were homogenized in SDS lysis buffer [Cell Signaling Technology (CST), #7722] and transferred onto polyvinylidene difluoride membranes. After blocking in 5% milk in tris-buffered saline and 0.1% Tween 20, membranes were incubated overnight with the following antibodies at 4°C: CASP9 (Abcam, #ab202068), cleaved CASP3 (CST, #9664), RIP3 (Millipore Sigma, #PRS2283), BAX (CST, #2772), BCL2 (CST, #3498), GSDMD (Santa Cruz Biotechnology, sc-393656), NLRP3 (CST, #15101), ACSL4 (Abcam, #ab155282), LC-3 (CST, #2775S), cGAS (CST, #31659), STING (CST, #13647S), TBK1 (CST, #38066), pTBK1 (CST, #5483), IRF3 (CST, #4302), pIRF3 (CST, #4947), p65 (CST, #8242), pp65 (CST, #3033), and GAPDH (CST, #5174). Membranes were incubated with horseradish peroxidase–conjugated secondary anti-rabbit or anti-mouse antibody (CST, #7074 and #7076) for 1 hour at room temperature. Signal was detected by enhanced chemiluminescence (SuperSignal West Femto Maximum Sensitivity Substrate, #34094; Thermo Fisher Scientific). Western blots were quantified using the Fiji software (*58*).

### Renal function and histology

BUN was determined by a TRACE DMA Urea kit (Thermo Electron Corporation, #TR12003), according to the manufacturer’s instructions. Electrolytes (Na, K, and Cl) and hemoglobin were measured with the iSTAT Portable Clinical Analyzer using i-STAT CHEM8+ cartridge (Abaxis, #10023291). Kidney tissue was stained with hematoxylin and eosin to investigate renal injury. The degree of tubulointerstitial damage was scored semiquantitatively on a 0 to 5+ scale, according to the percentage of the area affected by hydropic degeneration, hyaline casts, cytoplasmic vacuolization, loss of the brush border, and tubular lumen dilation (0 = normal, 1 = <10%, 2 = 10 to 25%, 3 = 26 to 50%, 4 = 51 to 75%, and 5 = >75%). Sirius red staining was performed according to the manufacturer’s protocol (Polysciences, #24901) to determine the degree of fibrosis. For each analysis, five to eight fields were randomly selected and quantified using the Fiji software (*58*). Each dot represents the mean score of a single animal.

### Primary culture of kidney tubule cells

Primary kidney tubule cells were isolated from 3- to 4-week-old mice. Kidneys were minced and incubated for 30 min at 37°C with collagenase I (Worthington Biochemical Product, #CLS-1). Digested kidney cells were filtered through the 100-, 70-, and 40-μm mesh to isolate single cells. Cell suspensions were cultured in RPMI 1640 (Corning, #10-040-CM) supplement with 10% fetal bovine serum (Atlanta Biologicals, #S11950), EGF (20 ng/ml; PeproTech #AF-100-15), insulin-transferrin-selenium (ITS) (Gibco, #51500-056), and 1% penicillin-streptomycin (Corning, #30-002-CI) at 5% CO_2_ and 37°C. Approximately 1 × 10^6^ cells were seeded per well in six-well plates and allowed to adhere overnight. Kidney tubule cells were treated with cisplatin (20 μM) for 8 hours in the presence and absence of BafA1 (100 nM; Sigma-Aldrich, #B1793).

### Cytotoxicity assay

Renal tubule cells from WT and *Casp9* HZ mice were seeded on 96-well plates (10,000 cells per well). After reaching 80% confluency, cells were treated with cisplatin (20 μM) for 8 hours. Live cells were monitored according to the manufacturer’s protocol (Promega, #G9200). Fluorescent signal at 400/505 nm (excitation/emission) was detected in a microplate reader (BioTek, Synergy H1).

### Cytosolic fraction extraction

Cytosolic, mitochondrial, and nuclear fractions from kidney tubules were extracted as described previously, by lysing cells with digitonin buffer (Thermo Fisher Scientific, #BN2006) (*59*). Cytosolic and mitochondrial DNAs were purified with the DNeasy Blood and Tissue Kit according to the manufacturer’s instructions (Qiagen, #69506). Quantitative PCR was performed using the ViiA 7 System (Life Technologies). The data were analyzed using the ΔΔ*C*_t_ method and shown as fold change. Primers are listed in table S4.

### Plasmid transfection

EGFP-LC3 (#11546), ptfLC3 (#21074), and Cox8-EGFP-mCherry (#78520) were purchased from Addgene. Plasmids were transfected with Lipofectamine 3000 according to the manufacturer’s protocol (Thermo Fisher Scientific, #L3000008). After transfection, tubule cells were incubated for 24 hours. The fluorescent signal was detected by a confocal microscope (Leica TCS SP8 Confocal). The number of puncta was counted in 10 cells, and mean puncta number were plotted.

### Immunostaining

Kidneys were fixed in 10% neutral formalin and 5-μm-thick sections were prepared. Sections were deparaffinized, followed by antigen retrieval in citrate buffer at 95°C for 10 min. Sections were allowed to cool slowly, washed in distilled water, and incubated in 3% H_2_O_2_ for 10 min. Sections were incubated in 5% goat serum (blocking buffer) at room temperature for 1 hour. In addition, avidin/biotin blocking was performed using the avidin/biotin blocking kit (Vector Laboratories, #004303). Sections were incubated overnight at 4°C with primary antibody for cleaved CASP3 (1:500; CST, #9664). After washing, sections were incubated with goat anti-rabbit secondary antibody for 1 hour at room temperature. ABC ready-to-use reagent (Vector Laboratories, #PK-6101) and ImmPACT DAB Peroxidase (HRP) Substrate (Vector Laboratories, #SK-4105) were used for visualization.

For immunofluorescence studies, sections were incubated with CASP9 (Novus, NB100-56118) antibody, washed with PBS, and incubated with donkey anti-mouse IgG (H + L) highly cross-adsorbed secondary antibody, Alexa Fluor 594 (Thermo Fisher Scientific, #A-21203) and fluorescein isothiocyanate-labeled LTL (Vector Laboratories, FL-1321-2), coverslip-mounted, and examined under a fluorescence microscope (OLYMPUS DP73).

### Caspase activity assay

Kidney tissue was homogenized in lysis buffer according to the manufacturer’s protocol (Biovision, #K118). Protein concentrations were measured using the Pierce BCA Protein Assay Kit (Thermo Fisher Scientific, #23225). Fifty micrograms of total protein was incubated at 37°C for 1 hour with reaction buffer (Biovision, #K118) and CASP3 or CASP9 substrate (Ac-DEVD-AFC: Biovision, #1007 and Ac-LEHD-AFC, Biovision #1075). The enzyme-catalyzed release of AFC was quantified in a fluorimeter (excitation of 400 nm and emission of 505 nm).

### Statistics

Data are presented as means ± SEM. Unpaired two-tailed Student’s *t* test was used for comparisons between two groups. One-way analysis of variance (ANOVA) with Tukey’s post hoc test was used to compare multiple groups. *P* < 0.05 was considered significant.

## Notes

View/request a protocol for this paper from *Bio-protocol*.
