# Supplementary material for: Genome-wide association studies identify the role of caspase-9 in kidney disease
Source: Sci Adv. 2021 Nov 5;7(45):eabi8051. doi: 10.1126/sciadv.abi8051 (PMC8570608; doi:10.1126/sciadv.abi8051)
Supplement: Supplementary file 1 — Figs. S1 to S11 Tables S1 to S4 [file sciadv.abi8051_sm.pdf]

Science Advances
AAAS

Supplementary Materials for

Genome-wide association studies identify the role of caspase-9 in kidney disease

Tomohito Doke, Shizheng Huang, Chengxiang Qiu, Xin Sheng, Matthew Seasock, Hongbo Liu, Ziyuan Ma, Matthew Palmer, Katalin Susztak*

*Corresponding author. Email: ksusztak@pennmedicine.upenn.edu

Published 5 November 2021, *Sci. Adv.* **7**, eabi8051 (2021)
DOI: 10.1126/sciadv.abi8051

This PDF file includes:

Figs. S1 to S11
Tables S1 to S4

Supplemental figure 1

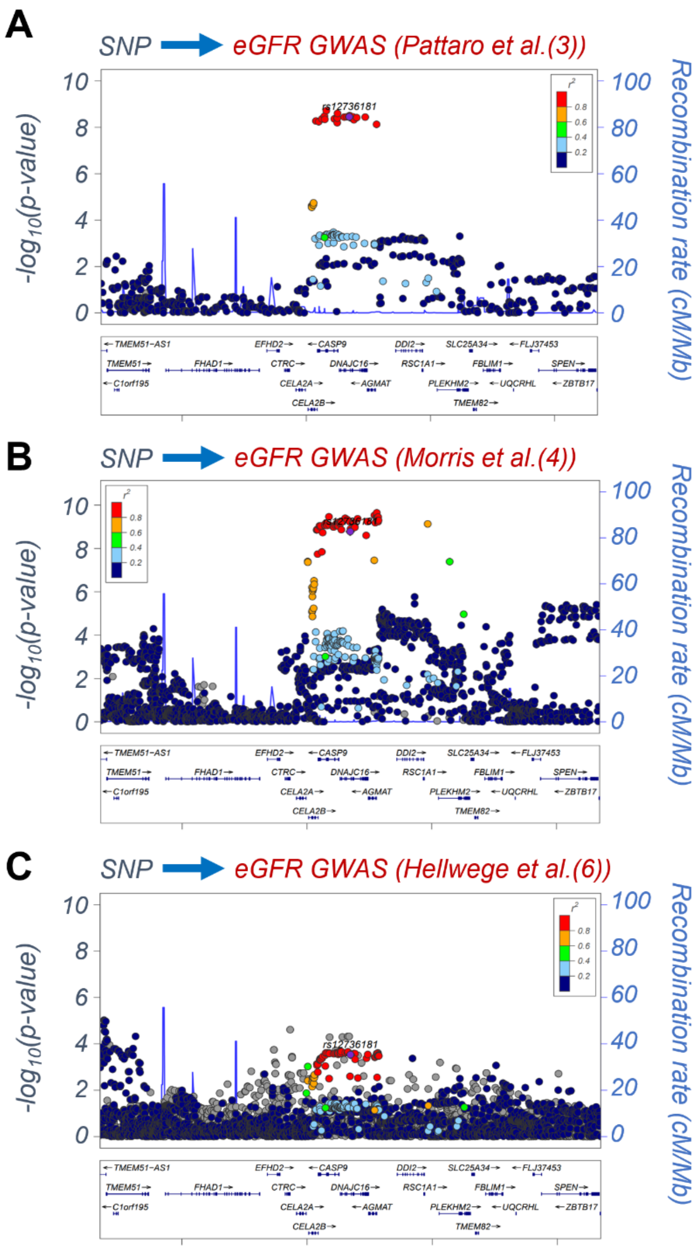

Supplemental Figure 1. Regional plots of genotype and eGFR (GWAS) in eGFR genome wide association studies (**A-C**) Locuszoom plots of chromosome 1 region of different eGFR GWAS (A; Pattaro et al (*3*), B; Morris et al (*4*), C; Hellwege et al (*6*)). X-axis shows the chromosomal location of SNPs. Y-axis shows the strength of association (-log10(p)).

Supplemental figure 2

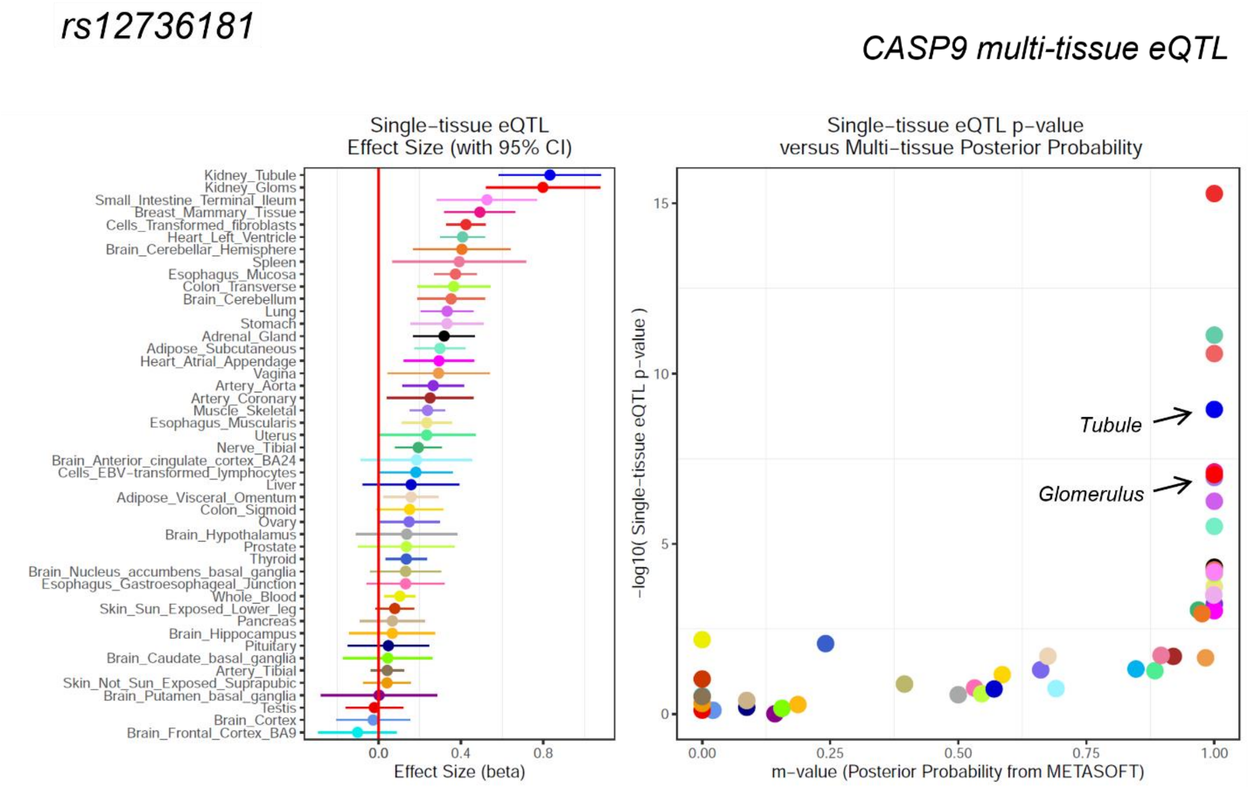

Supplemental Figure 2. Multi-tissue eQTL analysis (rs12736181) and *CASP9* expression in GTEx database
(Left) The effect size of single tissue eQTL with 95% confidence interval. The organs were arranged according to the observed effect sizes. (Right) Single-tissue eQTL p-value vs. multi-tissue posterior probability. Y axis; -log10 (single-tissue eQTL p-value). X axis; m-value (0-1), indicating posterior probability of eQTL effect in each tissue. M-value < 0.1 indicates no significant eQTL effect in tissue, while m-value > 0.9 indicates significant eQTL effect in tissue.

Supplemental figure 3

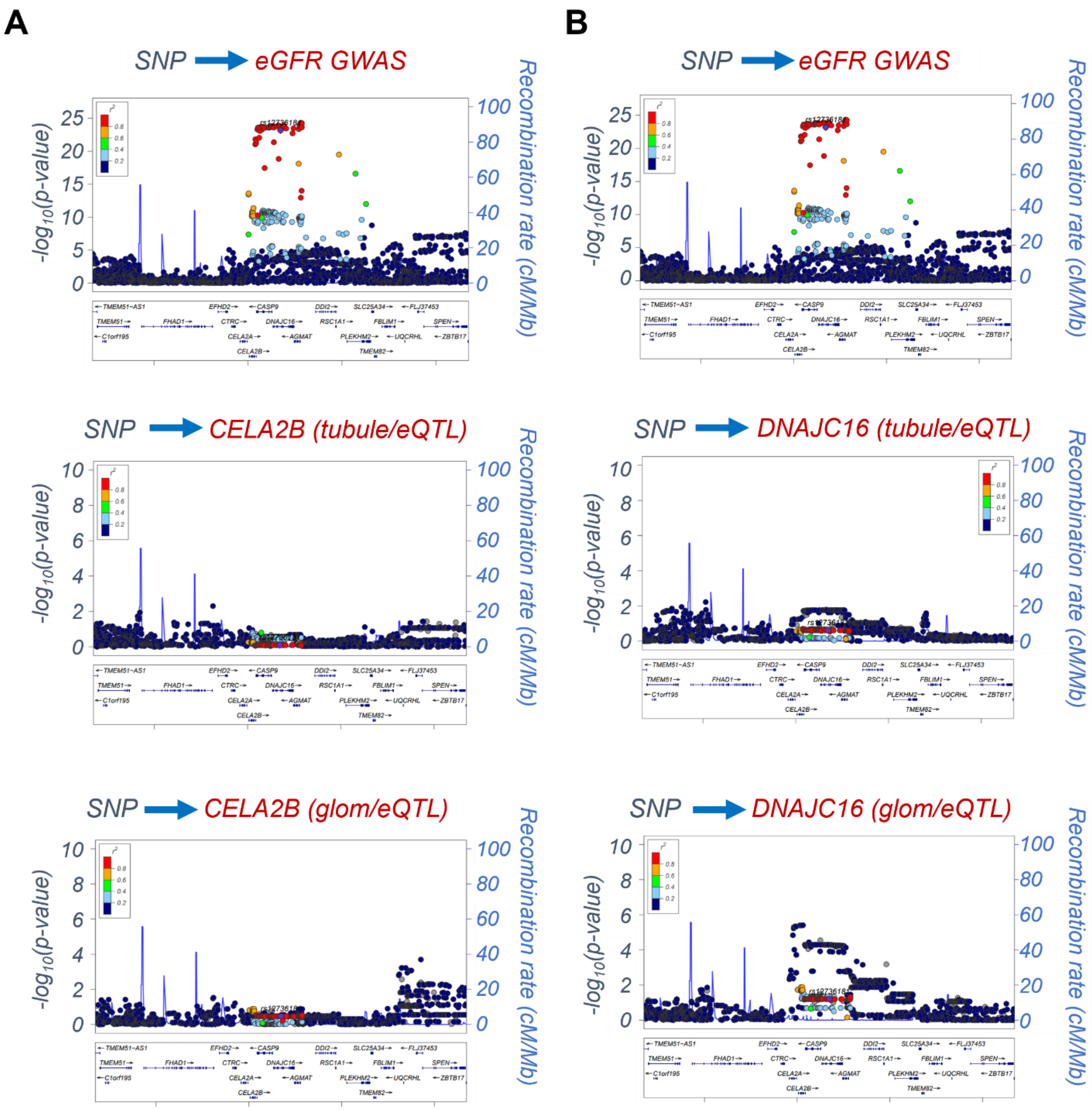

Supplemental Figure 3. Genotype-driven expression changes in *CELA2B* and *DNAJC16*
(**A**) LocusZoom plots of chromosome 1 region of CKD eGFR GWAS, *CELA2B* eQTL in kidney tubules and glomeruli.
(**B**) LocusZoom plots of chromosome 1 region of CKD eGFR GWAS, *DNAJ16* eQTL in kidney tubules and glomeruli.
X-axis; chromosomal location of SNPs. Y-axis; the strength of association (-log10(p)).

Supplemental figure 4

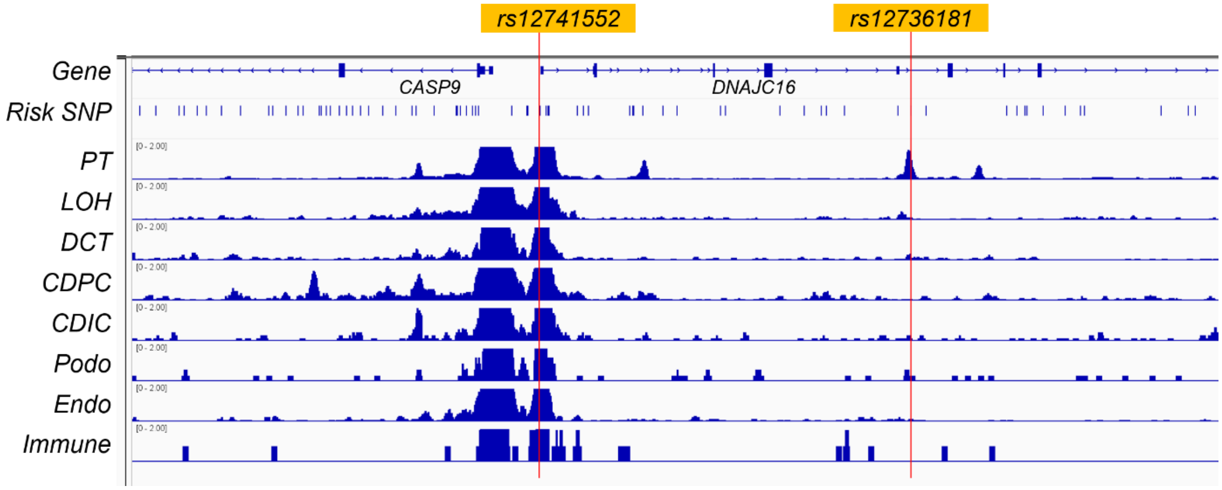

Supplemental Figure 4. Human kidney single nuclei open chromatin data and fine mapping of eGFR-associated GWAS variants

Human kidney single cell open chromatin atlas of eGFR GWAS chromosome 1 region. The 2 genetic variants (rs12741552 and rs12736181) with eGFR GWAS and tubule eQTL effects on *CASP9* overlapped with open chromatin area in human kidney cells were highlighted. The top row shows genome organization, followed by eGFR GWAS risk SNPs, open chromatin tracks in PT; proximal tubule, LOH; loop of Henle, DCT; distal convoluted tubule, CDPC; principal cells of the collecting duct, CDIC; intercalated cells of the collecting tubule, Podo; podocytes, Endo; endothelial, immune cell.

Supplemental figure 5

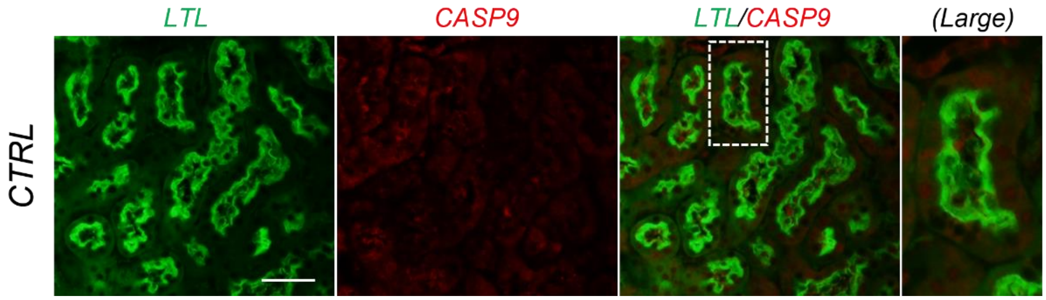

Supplemental Figure 5. Representative images of CASP9 and LTL staining in control mouse kidneys. Representative images of double immunostaining with CASP9 and proximal tubule marker, LTL (Lotus tetragonolobus lectin) in control kidneys. Scale bar:10 µm. The right panels are enlarged images of the boxed area.

Supplemental figure 6

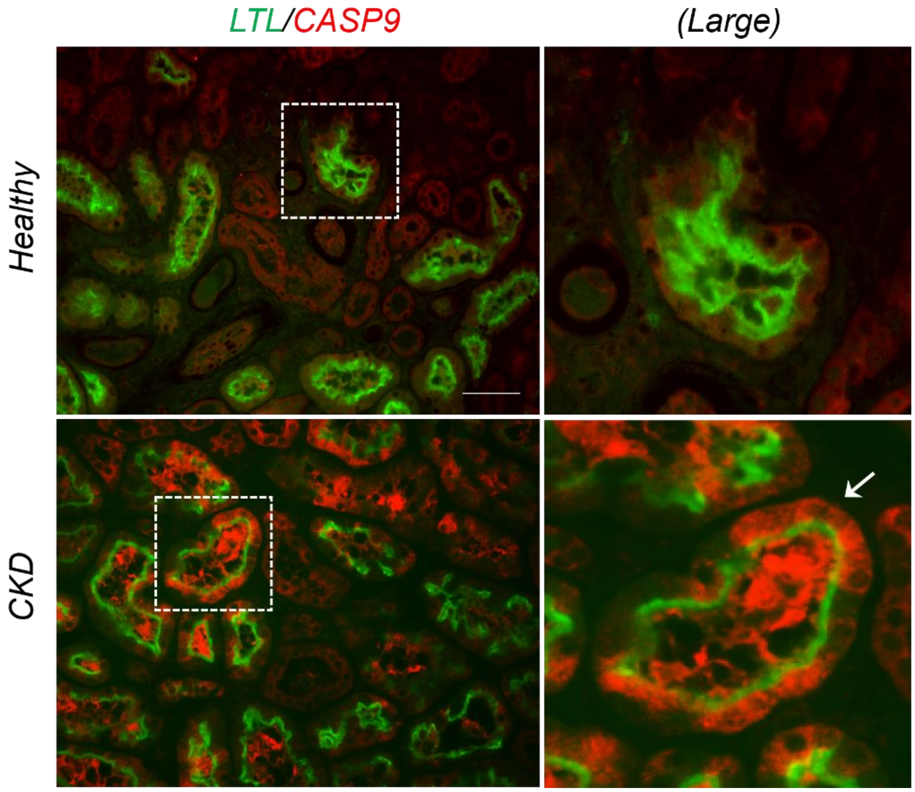

Supplemental Figure 6. Representative images of CASP9 and LTL staining in control and CKD human kidneys. Representative images of double immunostaining with CASP9 and proximal tubule marker, LTL (Lotus tetragonolobus lectin) in healthy (upper panel) and CKD (lower panel) human kidneys. Scale bar:20 μm. The right panels are enlarged images of the boxed areas. White arrow indicates CASP9 positive renal proximal tubules.

Supplemental figure 7

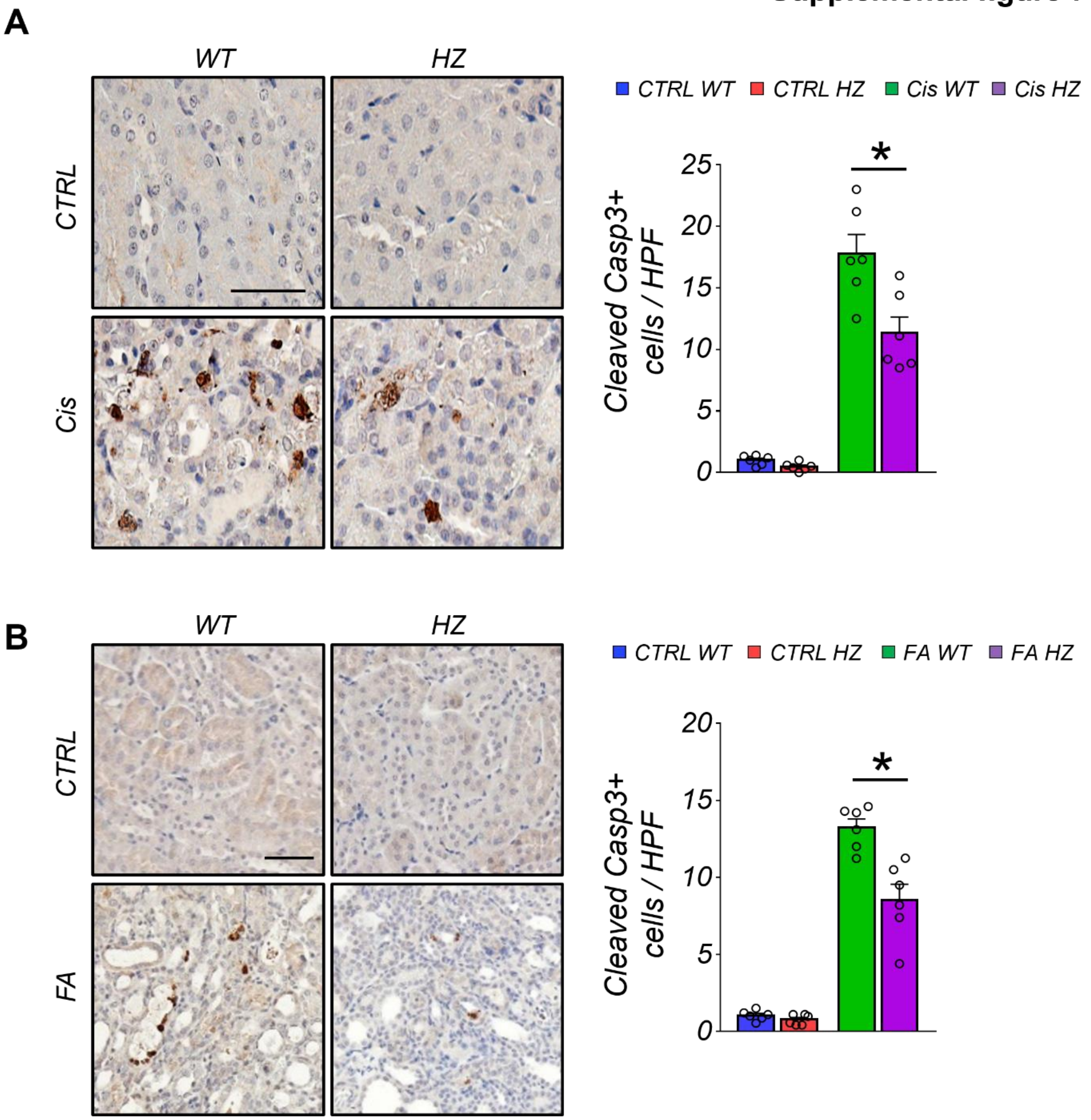

Supplemental Figure 7 Cleaved caspase-3 expression in mouse kidney disease models
(**A**-**B**) Representative cleaved caspase-3 staining and quantification of cleaved caspase-3 positive cells in kidneys of wild type (WT) and *Casp9* HZ (HZ) mice in control (CTRL) and cisplatin (Cis) (**A**) or folic acid (FA) (**B**) Bar; 20µm. Data are presented as the mean ± SEM. *$P < 0.05$.

Supplemental figure 8

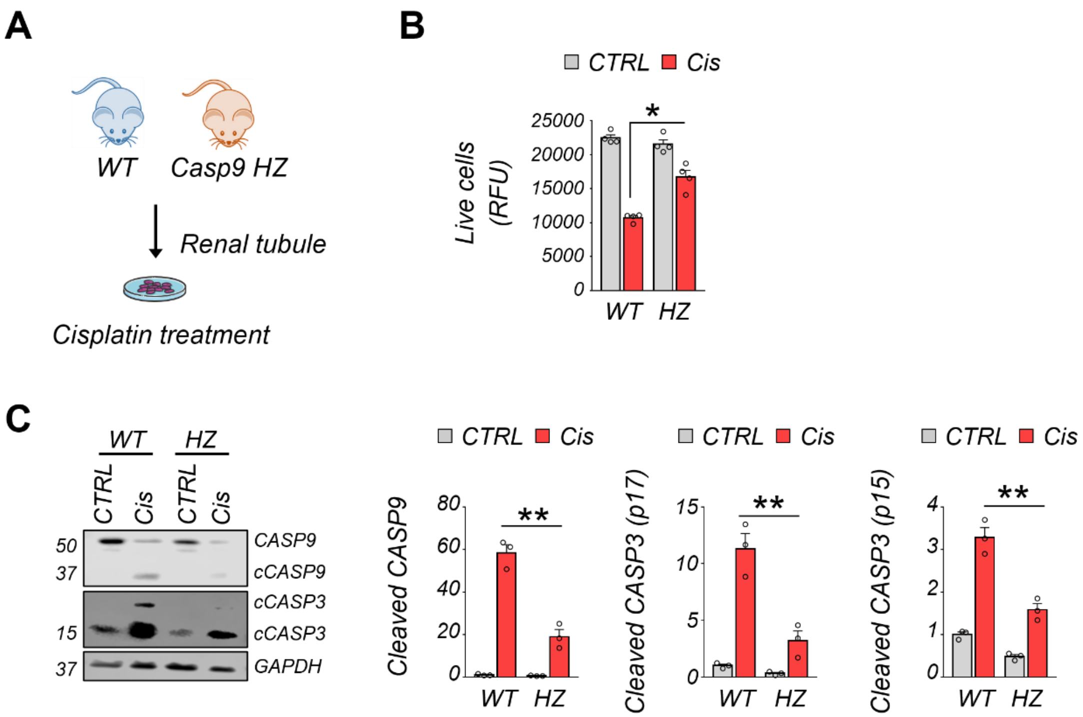

Supplemental Figure 8. Reduced apoptosis in *Casp9* HZ renal tubule cells

(**A**) Experimental design. The renal tubule cells were isolated form wild type (WT) and *Casp9* HZ (HZ) mice.

(**B**) Relative fluorescence unit (RFU)-based live cell analysis of cisplatin-(Cis) or PBS-treated (CTRL) renal tubule cells from wild type (WT) and *Casp9* HZ (HZ) mice. (n=3 independent experiments).

(**C**) Representative western blot image and quantification of pro-caspase-9 (CASP9), cleaved caspase-9 (cCSP9), cleaved caspase-3 (cCASP3) of cisplatin-(Cis) or PBS-treated (CTRL) renal tubule cells from wild type (WT) and *Casp9* HZ (HZ) mice. GAPDH was used for loading control. (n=3 independent experiment).

Data are presented as the mean ± SEM. *P < 0.05, **P < 0.01.

Supplemental figure 9

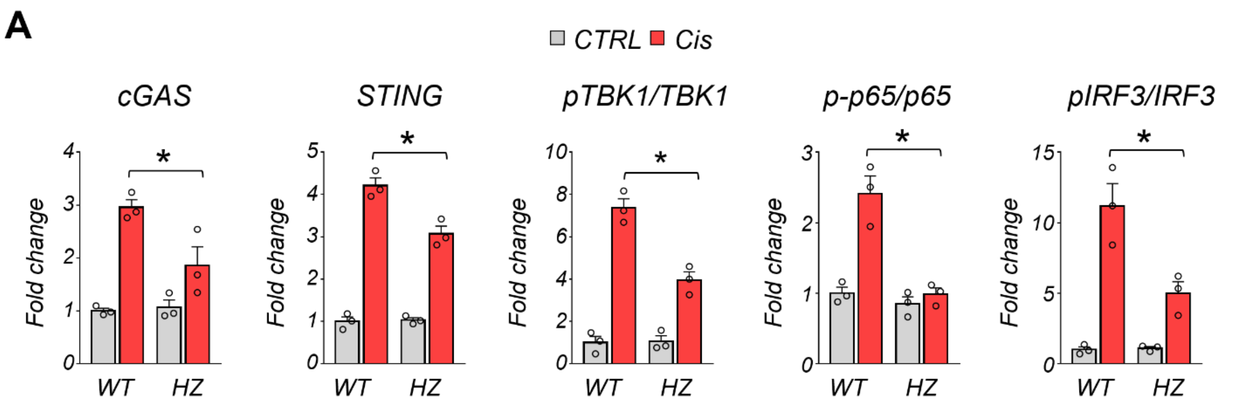

Supplemental Figure 9. Reduced protein expression of cytosolic nucleotide sensing pathways in cisplatin-treated *Casp9* HZ mice

(**A**) Quantification of cGAS, STING, pTBK1, pp65, and pIRF3 kidney western blots in control (CTRL) or cisplatin-treated (Cis) wild type (WT) and *Casp9* HZ (HZ) renal tubule cells. GAPDH was used for loading control. 3 independent experiment were performed. Data are presented as the mean ± SEM. *$P < 0.05$.

Supplemental figure 10

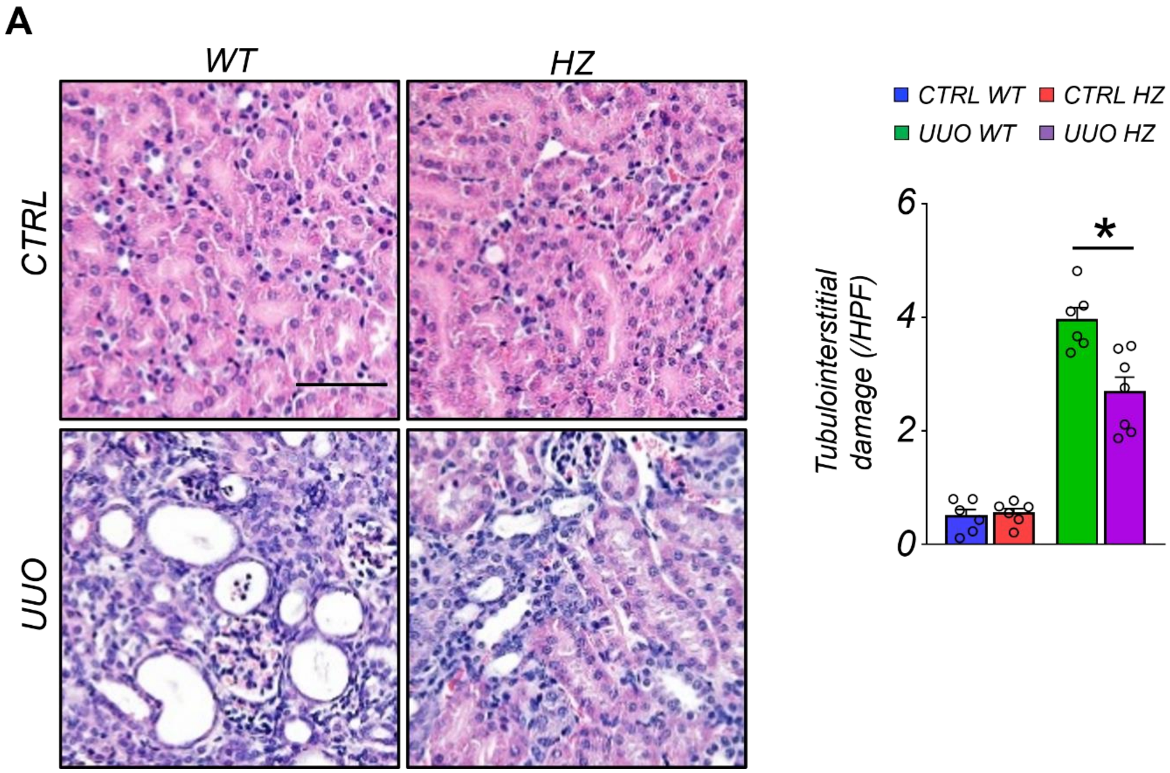

Supplemental Figure 10. Reduced tubulointerstitial injury in *Casp9* HZ mice subjected to UUO injury
(**A**) Representative Hematoxylin and Eosin stains of kidneys and quantification of renal tubule injury score in control (CTRL) and UUO-subjected kidney from WT (WT) and *Casp9* HZ (HZ) mice. Bar; 20µm. CTRL WT (n = 6), CTRL HZ (n = 6), UUO WT (n = 6), UUO HZ (n = 7). Data are presented as the mean ± SEM. *P < 0.05.

Supplemental figure 11

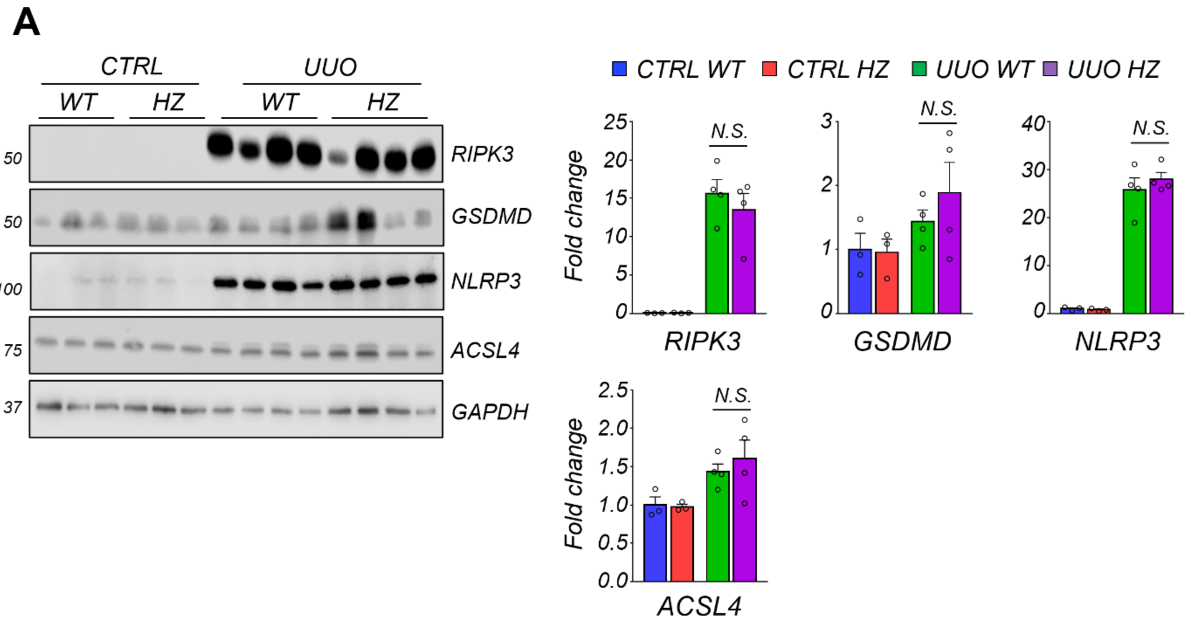

Supplemental Figure 11. Alteration in cell death pathway in WT and *Casp9* HZ mice subjected to UUO injury (A) Western blot image and quantification of RIPK3, gasdermin D (GSDMD), NLRP3, ACSL4 in control (CTRL) and UUO kidney from wild type (WT) and *Casp9* HZ (HZ) mice. GAPDH was used for loading control. Data are presented as the mean ± SEM. N.S. not significant.

Supplemental table 1. The result of the multi tissue eQTL analysis

Tissue	size	mvalue	pvalue	beta	beta_se	color
Kidney_Tubule	121	1	1.13E-09	0.83197	0.12524	blue2
Kidney_Gloms	119	1	9.29E-08	0.79799	0.13959	red
Adipose_Subcutaneous	298	1	3.07E-06	0.29805	0.06246	aquamarine2
Adipose_Visceral_Omentum	185	0.675	0.02005	0.15789	0.06716	bisque2
Adrenal_Gland	126	1	4.86E-05	0.31827	0.07507	black
Artery_Aorta	197	1	0.00059	0.26547	0.07567	blueviolet
Artery_Coronary	118	0.92	0.02029	0.2503	0.10605	brown
Artery_Tibial	285	0	0.30576	0.04229	0.04121	burlywood4
Brain_Anterior_cingulate_cortex_BA24	72	0.691	0.17926	0.18426	0.13528	cadetblue1
Brain_Caudate_basal_ganglia	100	0.156	0.68071	0.04511	0.10922	chartreuse1
Brain_Cerebellar_Hemisphere	89	0.976	0.00112	0.40455	0.11883	chocolate2
Brain_Cerebellum	103	1	5.89E-05	0.35253	0.08314	coral2
Brain_Cortex	96	0.021	0.77454	-0.02601	0.09048	cornflowerblue
Brain_Frontal_Cortex_BA9	92	0	0.29108	-0.102	0.09588	cyan2
Brain_Hippocampus	81	0.187	0.52635	0.06664	0.10455	darkgoldenrod1
Brain_Hypothalamus	81	0.5	0.2709	0.13682	0.12311	darkgray
Brain_Nucleus_accumbens_basal_ganglia	93	0.395	0.13175	0.13155	0.08627	darkkhaki
Brain_Putamen_basal_ganglia	82	0.142	0.98547	0.0026	0.14204	darkmagenta
Breast_Mammary_Tissue	183	1	7.72E-08	0.49195	0.0869	deeppink2
Cells_EBV-transformed_lymphocytes	114	0.847	0.04753	0.18109	0.09016	deepskyblue2
Cells_Transformed_fibroblasts	272	1	5.14E-16	0.42432	0.04858	firebrick2
Colon_Sigmoid	124	0.586	0.06968	0.15086	0.08229	gold1
Colon_Transverse	169	1	7.53E-05	0.36501	0.0893	greenyellow
Esophagus_Gastroesophageal_Junction	127	0.532	0.17183	0.13144	0.09554	hotpink1
Esophagus_Mucosa	241	1	2.58E-11	0.37346	0.05291	indianred2
Esophagus_Muscularis	218	1	0.00018	0.23427	0.06128	khaki2
Heart_Atrial_Appendage	159	1	0.00093	0.29341	0.08643	magenta
Heart_Left_Ventricle	190	1	7.41E-12	0.40792	0.05495	mediumaquamarine
Liver	97	0.57	0.18351	0.1582	0.11785	mediumblue
Lung	278	1	5.61E-07	0.333	0.06472	mediumorchid2
Muscle_Skeletal	361	1	1.13E-07	0.23781	0.04381	mediumpurple2
Nerve_Tibial	256	0.969	0.00088	0.19284	0.05717	mediumseagreen
Ovary	85	0.661	0.05028	0.14824	0.0743	mediumslateblue
Pancreas	149	0.087	0.39912	0.06704	0.07921	navajowhite3
Pituitary	87	0.087	0.63389	0.04759	0.09946	navy
Prostate	87	0.546	0.25388	0.13472	0.11704	olivedrab1
Skin_Not_Sun_Exposed_Suprapubic	196	0	0.48388	0.04112	0.05859	orange2
Skin_Sun_Exposed_Lower_leg	302	0	0.09375	0.07866	0.04677	orangered3
Small_Intestine_Terminal_Ileum	77	1	6.82E-05	0.52643	0.12215	orchid1
Spleen	89	0.896	0.01896	0.39168	0.16289	palevioletred2
Stomach	170	0.999	0.00032	0.33246	0.0899	plum2
Testis	157	0	0.77064	-0.02062	0.07056	red2
Thyroid	278	0.241	0.00854	0.13443	0.05069	royalblue3
Uterus	70	0.884	0.05344	0.2337	0.11809	seagreen2
Vagina	79	0.983	0.02232	0.2911	0.12399	tan2
Whole_Blood	338	0	0.00655	0.10378	0.0379	yellow2

Supplemental table 2. Characteristics of control and aging mice of wild type (WT) and Casp9 HZ (HZ) mice

	CTRL (6 months)		Aging (24 months)	
	n = 6	n = 6	n = 4	n = 6
	WT	HZ	WT	HZ
Body weight (g)	27.0 ± 0.7	27.1 ± 0.8	30.3 ± 0.6^{A}	30.0 ± 1.6^{B}
BUN (mg/dl)	24.1 ± 1.2	23.5 ± 1.6	21.8 ± 2.0	25.4 ± 1.0
Na (mmol/L)	150 ± 0.4	149 ± 0.3	151 ± 0.5	149 ± 1.0
K (mmol/L)	5.3 ± 0.2	4.8 ± 0.1	5.4 ± 0.2	4.7 ± 0.2
Cl (mmol/L)	115.6 ± 1.7	115.5 ± 2.8	116.3 ± 4.0	112.4 ± 1.7
Glu (mg/dl)	180 ± 10.5	167 ± 15.2	140 ± 5.3	176 ± 10.1
Hb (g/dL)	13.2 ± 0.7	13.3 ± 0.6	12.2 ± 1.0	12.4 ± 0.4

Data are presented as the mean ± SEM. Statistical significance was determined by 1-way ANOVA followed by Tukey’s post hoc test. CTRL: control, BUN: blood urea nitrogen, Glu: glucose, Hb; hemoglobin.
A; $p < 0.05$ vs CTRL WT
B; $p < 0.05$ vs CTRL HZ

Supplemental table 3. Guide RNA sequences and target deleted region

		Sequence	position	START	END	Target SNP
oligo1	Fw	5'-CACCGAAACGCCTCTCCCATACGGG-3’	Chr1	15868740	15868759	rs12736181
	Rv	5’-AAACCCCGTATGGGAGAGGCGTTTC-3’	Chr1	15868740	15868759	rs12736181
oligo2	Fw	5'-CACCGGTCCTGAATGCGGCCTACCT-3’	Chr1	15869787	15869806	rs12736181
	Rv	5'-AAACAGGTAGGCCGCATTCAGGACC-3’	Chr1	15869787	15869806	rs12736181
oligo1	Fw	5'-CACCGATTCGTAGAAGTGAACGAAT-3'	Chr1	15852423	15852442	rs12741552
	Rv	5'-AAACATTCGTTCACTTCTACGAATC-3'	Chr1	15852423	15852442	rs12741552
oligo2	Fw	5'-CACCGAGTGCAAGTTTAATGCCGCC-3'	Chr1	15854245	15854264	rs12741552
	Rv	5'-AAACGGCGGCATTAAACTTGCACTC-3'	Chr1	15854245	15854264	rs12741552

Supplemental table 4. Primer information for QRT-PCR

gene	Forward (5’-3’)	Reverse (5’-3’)
Apaf1	GCAAACGAGAGGAAAAGCATTAA	GCAGACCAGGAACAACACTTCA
Bax	CTGCAGAGGATGATTGCTGA	GAGGAAGTCCAGTGTCCAGC
Casp9	GTCAAGTTTGCCTACCCCCA	GAGCCCACTGCTCCAGAATG
Col1a1	TGACTGGAAGAGCGGAGAGT	GTTCGGGCTGATGTACCAGT
Col3a1	ACAGCTGGTGAACCTGGAAG	ACCAGGAGATCCATCTCGAC
Csf2	GGCCTTGGAAGCATGTAGAGG	GGAGAACTCGTTAGAGACGACTT
Cxcl10	AAGTGCTGCCGTCATTTTCT	GTGGCAATGATCTCAACACG
Fn1	CCGTGTAAGGGTCAAAGCAT	ACAAGGTTCGGGAAGAGGTT
Gapdh	ATGTTTGTGATGGGTGTGAA	ATGCCAAAGTTGTCATGGAT
Havcr1	TCCACACATGTACCAACATCAA	GTCACAGTGCCATTCCAGTC
Icam1	GCCTCCGGACTTTCGATCTT	TAGGAGATGGGTTCCCCCAG
Il1b	CCCTGCAGCTGGAGAGTGTGGA	TGTGCTCTGCTTGTGAGGTGCTG
Mlkl	GAAGACAGACCTAGACAGCGG	CCAGTAGCTTCACCACTCGAC
Ripk3	AATTGTACTCTGGGAAATTGCCA	TCTCCAAGATTCCGTCCACAG
Slc12a1	GGAGTTGTGAAGTTTGGATGGG	ATTCCCGCTTCTCCTACAATCC
slc22a30	CTAGGCATGGTGGCAGTGCT	CCGAGCCGACTCTGACATCC
slc27a2	CGCTGACATCGTGGGACTGG	TCGACCCTCATGACCTGGCA
Tnfa	TGTGAGGAAGGCTGTGCATT	GGTCAGGTTGCCTCTGTCTC
Vim	GATCGATGTGGACGTTTCCAA	ATACTGCTGGCGCACATCAC
hCASP9	GCTTCTCCTCGCTGCATTTC	GCAGCTGGTCCCATTGAAGA
hDNAJC16	TTGGTGCTTTAGCTGCATTCA	CGCCAATTCCTACACCCAATTC
hCELA2B	GCAGCACCGTGAAGACGAATA	ATGCCTGACAGTTCAGCGG
hGAPDH	CTGGGCTACACTGAGCACC	AAGTGGTCGTTGAGGGCAATG
